# Inorganic polyphosphate and the stringent response coordinately control cell division and cell morphology in *Escherichia coli*

**DOI:** 10.1128/mbio.03511-24

**Published:** 2024-12-27

**Authors:** Christopher W. Hamm, Michael J. Gray

**Affiliations:** 1 Department of Microbiology, Heersink School of Medicine, University of Alabama at Birmingham, Birmingham, Alabama, USA; Massachusetts Institute of Technology, Cambridge, Massachusetts, USA

**Keywords:** (p)ppGpp, polyphosphate, cell division, cell morphology, stringent response, stress response

## Abstract

Bacteria encounter numerous stressors in their constantly changing environments and have evolved many methods to deal with stressors quickly and effectively. One well-known and broadly conserved stress response in bacteria is the stringent response, mediated by the alarmone (p)ppGpp. (p)ppGpp is produced in response to amino acid starvation and other nutrient limitations and stresses and regulates both the activity of proteins and expression of genes. *Escherichia coli* also makes inorganic polyphosphate (polyP), an ancient molecule evolutionary conserved across most bacteria and other cells, in response to a variety of stress conditions, including amino acid starvation. PolyP can act as an energy and phosphate storage pool, metal chelator, regulatory signal, and chaperone, among other functions. Here we report that *E. coli* lacking both (p)ppGpp and polyP have a complex phenotype indicating previously unknown overlapping roles for (p)ppGpp and polyP in regulating cell division, cell morphology, and metabolism. Disruption of either (p)ppGpp or polyP synthesis led to the formation of filamentous cells, but simultaneous disruption of both pathways resulted in cells with heterogenous cell morphologies, including highly branched cells, severely mislocalized Z-rings, and cells containing substantial void spaces. These mutants also failed to grow when nutrients were limited, even when amino acids were added. These results provide new insights into the relationship between polyP synthesis and the stringent response in bacteria and point toward their having a joint role in controlling metabolism, cell division, and cell growth.

**IMPORTANCE:**

Cell division is a fundamental biological process, and the mechanisms that control it in *Escherichia coli* have been the subject of intense research scrutiny for many decades. Similarly, both the (p)ppGpp-dependent stringent response and inorganic polyphosphate (polyP) synthesis are well-studied, evolutionarily ancient, and widely conserved pathways in diverse bacteria. Our results indicate that these systems, normally studied as stress-response mechanisms, play a coordinated and novel role in regulating cell division, morphology, and metabolism even under non-stress conditions.

## INTRODUCTION

Bacteria have evolved stress response systems to ensure their survival in ever-changing environments and against host defenses. Two widely conserved stress response strategies are the general stress response in bacteria known as the stringent response, and the nearly universally conserved polyphosphate (polyP) pathway (1–4). However, the extent and nature of the interactions between these stress response pathways is poorly understood, as are their roles in bacterial physiology under non-stress conditions.

The stringent response is mediated by the small molecule guanosine 5′-diphosphate 3′-diphosphate (ppGpp) and guanosine 5′-triphosphate 3′-diphosphate (pppGpp), known collectively as (p)ppGpp (5–7). In *E. coli,* (p)ppGpp is synthesized by the proteins RelA and SpoT (8–12), part of the RSH (**R**elA-**S**poT **h**omolog) family, with SpoT being able to both synthesize and degrade (p)ppGpp (8, 9, 13–17). SpoT is an essential gene in *relA*^+^ strains, as (p)ppGpp levels quickly rise to toxic levels in the absence of (p)ppGpp hydrolysis (18–22). (p)ppGpp can bind to and regulate the activity of proteins, including binding RNA polymerase and DksA to regulate genome-wide gene expression (23–25). (p)ppGpp downregulates DNA and RNA synthesis, protein production, and cell growth and modifies gene expression of up to one-third of the genome in *E. coli* in response to a wide variety of stressors (5, 6, 14, 26–30). In recent years, (p)ppGpp has emerged as a master regulator of bacterial cellular biology and is now known to affect nearly every aspect from growth rate, sporulation, motility, competence, biofilm formation, toxin production, and virulence of pathogenic bacteria (6, 27, 31–36). Relevant to the results we present here, the stringent response has also been linked to the regulation of cell division, albeit by currently unknown mechanisms (37–39).

Inorganic polyphosphate (polyP) is an evolutionarily ancient biopolymer and is found in nearly all bacterial and many eukaryotic cells (4, 40–44). In bacteria, polyP is involved in regulating gene expression, chelation of metals, acting as a protein-stabilizing chaperone, and can affect biofilm formation, stress sensing, quorum sensing, and motility (3, 4, 45–52). PolyP chains can be from dozens to thousands of phosphates long and are produced by polyphosphate kinase (PPK) and degraded by the enzyme exopolyphosphatase (PPX) (53–55). Pathogenic bacteria lose their virulence when polyP production is abolished, suggesting that polyP is vital for the ability of bacteria to cause harm, and motivating ongoing searches for PPK inhibitors as antivirulence drugs (4, 51, 56–59).

PolyP and the stringent response have long been suspected to be linked together, affecting virulence and the ability of bacteria to survive. Both *relA spoT* mutants lacking (p)ppGpp and *ppk* mutants lacking polyP have multiple amino acid auxotrophies and growth defects on minimal media, for example, suggesting parallel or linked roles in surviving nutritional stresses (48, 60, 61). (p)ppGpp is known to play a role in preventing the degradation of polyP in bacteria by inhibiting PPX (43, 44, 62, 63), but evidence also suggests other potential links between these two fundamental systems (43, 64). Recent work from our laboratory (42) showed that (p)ppGpp is not itself required for polyP synthesis, but did find a link between the production of polyP in *E. coli* and the transcription factor DksA, which acts in coordination with (p)ppGpp to regulate gene expression within the cell (63, 65–67). Mutations of RNA polymerase that mimic the effects of (p)ppGpp binding to that enzyme on transcription (*a.k.a*. stringent alleles) (68–72) also reduce polyP synthesis by an unknown mechanism (43).

While the effects of (p)ppGpp and polyP during stress have been the focus of many investigations, their effect on cell physiology during normal growth conditions remains poorly understood, and the interaction between these two fundamental systems is not clear (6, 8, 13, 36, 43, 73). In this work, we identify striking and unexpected combinatorial phenotypes in *E. coli* mutants lacking both (p)ppGpp and polyP that suggest that these two pathways coordinately regulate fundamental mechanisms of cell division and morphology, even in the absence of nutritional stress. This provides new insights into the roles of general stress response pathways under non-stress conditions and raises important questions about the mechanisms bacteria use to maintain their integrity during growth, highlighting gaps in our knowledge in an area that has been the subject of many decades of research scrutiny.

## RESULTS

### Triple mutants lacking *ppk*, *relA*, and *spoT* have a growth defect on a minimal medium that cannot be rescued with casamino acids

**Fig 1:**
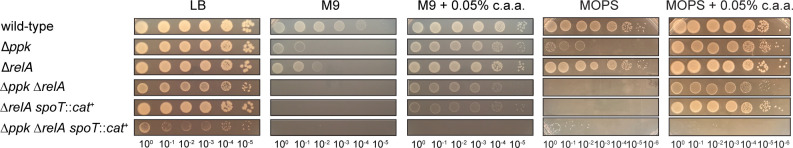
Triple mutants lacking *ppk*, *relA*, and *spoT* have a growth defect on a minimal medium that cannot be rescued with casamino acids. *E. coli* strains MG1655 (wild type), MJG0224 (MG1655 ∆*ppk-749*), MJG0226 (MG1655 ∆*relA782*), MJG1116 (MG1655 ∆*ppk-749* ∆*relA782*), MJG1136 (MG1655 ∆*relA782 spoT207::cat*^+^), and MJG1137 (MG1655 ∆*ppk-749* ∆*relA782 spoT207::cat*^+^) were grown overnight in LB broth, then rinsed and normalized to an A_600_ = 1 in PBS. Aliquots (5 µL) of serially diluted suspensions were spotted on LB, M9 glucose, M9 glucose containing 0.05% (wt/vol) casamino acids (c.a.a.), MOPS glucose, or MOPS glucose containing 0.05% (wt/vol) casamino acids (c.a.a.) plates and incubated overnight at 37°C (representative image from at least three independent experiments).

While both *ppk* and *relA spoT* mutants have well-described amino acid requirements in minimal media (44, 60, 74–76), both grow robustly on rich LB medium (Fig. 1). We were surprised, therefore, to find that a triple *ppk relA spoT* mutant, entirely lacking the ability to synthesize both polyP and (p)ppGpp (74, 77), has a substantial growth defect on LB (Fig. 1). Even more surprisingly, while we could easily rescue the growth defects of *ppk*, *relA*, *ppk relA*, and *relA spoT* mutants on minimal media by adding 0.05% (wt/vol) casamino acids (an undefined mixture of amino acids generated by acid hydrolysis of casein), casamino acids were unable to restore growth of the *ppk relA spoT* triple mutant on minimal medium. This was true both on phosphate-buffered M9 minimal medium (69.5 mM P_i_) (78) and on MOPS minimal medium (1.23 mM P_i_) (79). Growth of the mutant strains was not affected by plating on hypo-osmotic LBK (LB medium with no added NaCl) or hyper-osmotic LBK +500 mM sucrose (Fig. S1A). This indicated to us that there was an amino acid- and osmolarity-independent defect in the *ppk relA spoT* strain that was not present in either parent strain, suggesting a previously unsuspected redundant metabolic role for polyP and (p)ppGpp in *E. coli*.

*E. coli* lacking (p)ppGpp have low levels of the general stress response sigma factor RpoS (80, 81), and polyP has also been reported to increase RpoS expression (82), so we tested whether the phenotype of a *ppk relA spoT* mutant was also present in a *ppk rpoS* mutation, but it was not (Fig. S1B) indicating that the role of (p)ppGpp in a *ppk* mutant is independent of RpoS-dependent transcriptional regulation (81). We also quantified guanosine nucleotide pools in *E. coli* in minimal media and found that (p)ppGpp accumulation was significantly higher in ∆*ppk* cells than in *ppk*^+^ strains (Fig. S2A and B), supporting the idea that these two molecules can in some way compensate for each other’s absence.

### The growth defect of a *ppk relA spoT* mutant on a minimal medium with casamino acids can be rescued by the expression of either PPK or synthetase-active SpoT

**Fig 2:**
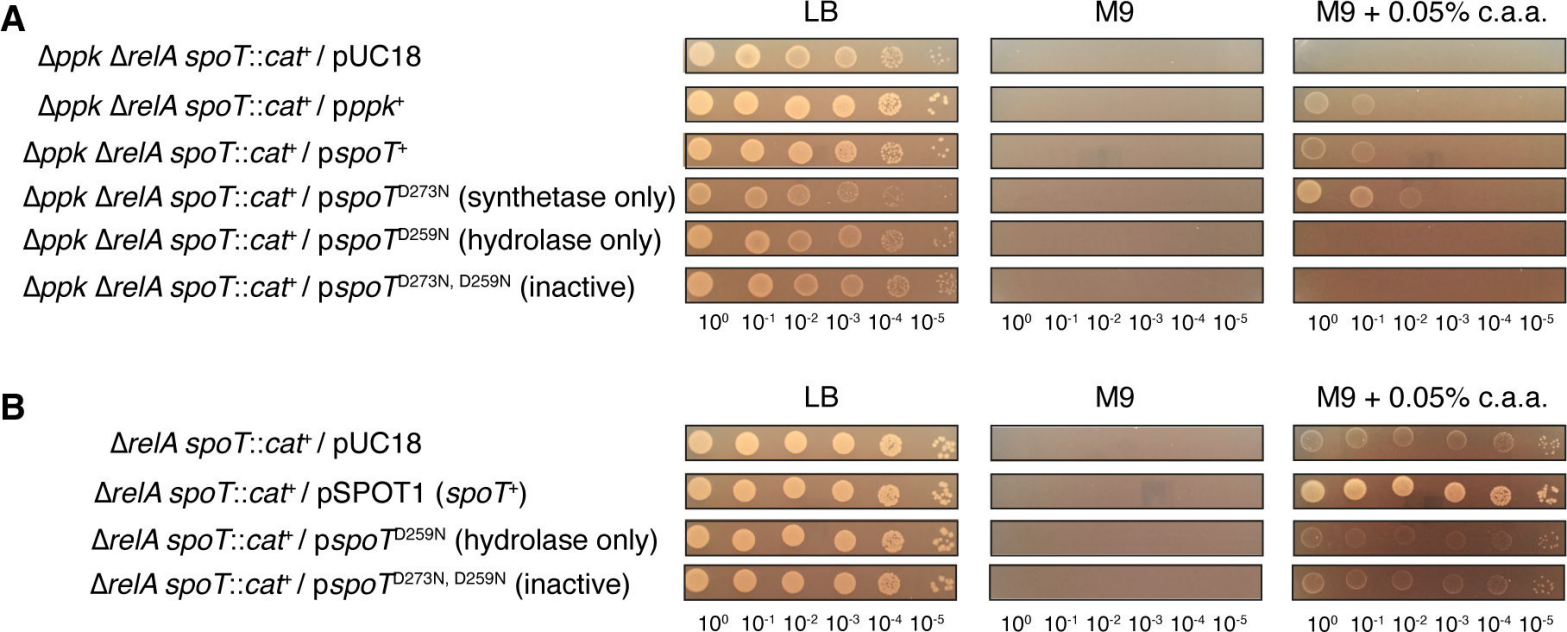
The growth defect of a *ppk relA spoT* mutant on a minimal medium with casamino acids can be rescued by expression of either PPK or synthetase-active SpoT. *E. coli* strains (**A**) MJG1137 (MG1655 ∆*ppk-749* ∆*relA782 spoT207::cat*^+^) or (**B**) MJG1136 (MG1655 ∆*relA782 spoT207::cat*^+^) containing the indicated plasmids (p*ppk*^+^ =pPPK8, p*spoT*^+^ =pSPOT1, p*spoT*^D73N^ = pSPOT2, p*spoT*^D259N^ = pSPOT3, p*spoT*^D73N, D259N^ = pSPOT4) were grown overnight in LB broth containing ampicillin, then rinsed and normalized to an A_600_ = 1 in PBS. Aliquots (5 µL) of serially diluted suspensions were spotted on LB, M9 glucose, or M9 glucose containing 0.05% (wt/vol) casamino acids (c.a.a.) plates containing ampicillin and incubated overnight at 37°C (representative image from at least three independent experiments).

Growth of the *ppk relA spoT* mutant on a minimal medium containing casamino acids was restored by ectopic expression of either PPK or SpoT (Fig. 2A). Complementation by SpoT was dependent on (p)ppGpp synthesis, since expression of a SpoT^D73N^ mutant allele lacking (p)ppGpp hydrolase activity (83) restored growth, but the expression of SpoT^D259N^ and SpoT^D73N, D259N^ alleles, which lack (p)ppGpp synthetase activity (83), did not. Expression of wild-type SpoT enhanced the growth of a *relA spoT* mutant on a minimal medium containing casamino acids (Fig. 2B), but expression of SpoT alleles lacking (p)ppGpp synthetase activity did not, suggesting that (p)ppGpp synthesis underlies this effect. The *relA spoT* mutant could not be transformed with a plasmid encoding hydrolase-defective SpoT^D73N^ (data not shown). This was unsurprising since accumulation of (p)ppGpp in *E. coli* cells lacking (p)ppGpp hydrolase activity is well known to prevent growth (74–76). The more surprising result from this experiment is that the *ppk relA spoT* mutant did tolerate SpoT^D73N^ expression (Fig. 2A), suggesting that polyP is somehow involved in (p)ppGpp-dependent growth inhibition.

To further probe the effect of polyP on the phenotype of these strains, we added exogenous polyP to the growth media, which had no effect on the growth of any tested strain (Fig. S3A), indicating that polyP must be produced intracellularly to impact these phenotypes. We also expressed the hyperactive PPK^E245K^ variant (84, 85) from a plasmid to enhance polyP levels above natural levels (Fig. S3B). This rescued the growth defects of *ppk*, *ppk relA*, and, surprisingly, *relA spoT* mutants on MOPS glucose without amino acids as well as partially restoring growth to the *ppk relA spoT* mutant on MOPS glucose with 0.05% casamino acids (Fig. S3C), similar to the effect of the wild-type *ppk* plasmid in Fig. 2A.

### Both tryptone and yeast extract contain components that rescue the growth defect of a *ppk relA spoT* mutant on a minimal medium with casamino acids

**Fig 3:**
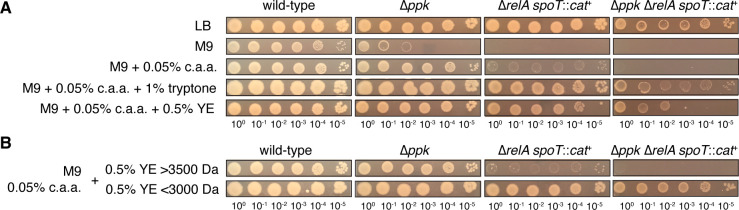
Both tryptone and yeast extract contain components that rescue the growth defect of a *ppk relA spoT* mutant on a minimal medium with casamino acids. *E. coli* strains MG1655 (wild type), MJG0224 (MG1655 ∆*ppk-749*), MJG1136 (MG1655 ∆*relA782 spoT207::cat*^+^), and MJG1137 (MG1655 ∆*ppk-749* ∆*relA782 spoT207::cat*^+^) were grown overnight in LB broth, then rinsed and normalized to an A_600_ = 1 in PBS. Aliquots (5 µL) of serially diluted suspensions were spotted on LB, M9 glucose, or M9 glucose containing the indicated percentages (wt/vol) of casamino acids (c.a.a.) and (**A**) tryptone or yeast extract (YE) or (**B**) yeast extract fractions containing compounds either greater than 3,500 Da or less than 3,000 Da and incubated overnight at 37°C (representative image from at least three independent experiments).

LB medium consists of 1% (wt/vol) of tryptone (a tryptic digest of casein), 0.5% (wt/vol) of yeast extract (prepared from the soluble fraction of boiled *Saccharomyces cerevisiae* cells), and 0.5% (wt/vol) of NaCl (78). Both tryptone and yeast extract at these concentrations restored growth of the *ppk relA spoT* triple mutant on a minimal medium containing casamino acids (Fig. 3A). The same effect could be seen in liquid media and with strains containing a complete deletion of *spoT* rather than the *spoT207::cat*^+^ insertion mutation (Fig. S4). Those experiments also showed that growth of all of the tested mutant strains in minimal media supplemented with a defined mixture of purified amino acids (yeast synthetic dropout mix supplement; Sigma Aldrich cat. #Y1501) was comparable to that in media supplemented with casamino acids, supporting our conclusions from Fig. 3A. However, we found that variability was high in liquid media, especially later in the growth curves (possibly due to unpredictable accumulation of suppressor mutations), and opted to continue using solid media to evaluate the growth phenotype of the *ppk relA spoT* mutant.

We used filtration and dialysis to divide yeast extract into fractions with molecular weights greater than 3,500 Da or less than 3,000 Da and found that the component(s) responsible for restoring growth of the *ppk relA spoT* mutant on minimal medium containing casamino acids were present in the small molecule (e.g., less than 3,000 Da) fraction (Fig. 3B). Because LB medium is regularly sterilized by autoclaving, the compound(s) in question must also be heat-stable, and we are currently working to identify the relevant molecule(s), which we expect to give insights into the metabolic pathway(s) responsible for the inability of the *ppk relA spoT* mutant to grow on minimal media.

### Morphological defects of *ppk relA spoT* cells 1: filamentation

**Fig 4:**
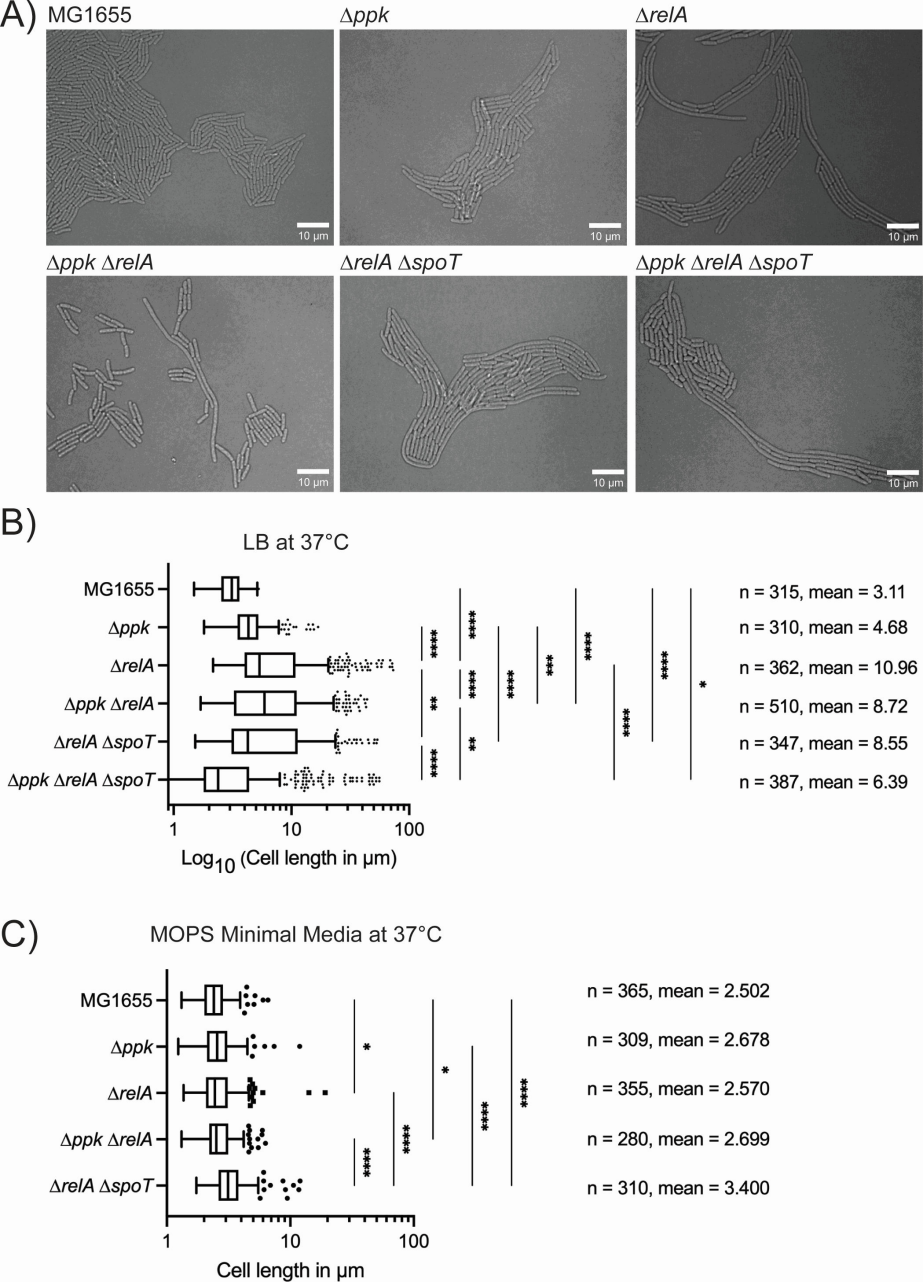
Microscopy of polyP and (p)ppGpp mutants shows filamentous cell growth. (**A**) Confocal microscopy images of MG1655 (MJG0001), *∆ppk* (MJG0224), *∆relA* (MJG0226), *∆ppk ∆relA* (MJG1116), *∆relA ∆spoT* (MJG1287), and *∆ppk ∆relA ∆spoT* (MJG1282). Images were captured on LB agarose pads incubated at 37°C and imaged every 5 minutes while growing. (**B**) Length of cells growing at 37°C on an LB agarose pad and (**C**) length of cells growing at 37°C on a MOPS minimal media agarose pad. Length of cells was determined and manually calculated using FIJI using the line tool and measuring length with built-in measurements to FIJI after setting the appropriate scale. *n* = number of cells. Statistical significance was calculated in Prism GraphPad by one-way ANOVA, *P-value <0.05, **P-value <0.005, ***P-value <0.0005, ****P-value <0.0001.

Since the *ppk relA spoT* mutant did not grow as well on LB plates as the other mutant strains, we wanted to see whether there were visible morphological defects present in this strain or its parent strains. We performed confocal time-lapse microscopy and noted that cells lacking either polyP or (p)ppGpp grown in LB are longer than wild-type cells, indicating defects in cell division (Fig. 4). Our results showed that a *ppk* mutant lacking polyP was slightly longer than the wild type MG1655 while growing in exponential phase on LB (Fig. 4). Strains deficient in (p)ppGpp were also filamentous, with a *relA* mutant being longer on average than a double *relA spoT* mutant (Fig. 4). The *ppk relA* mutant lacking polyP and only containing SpoT for production of (p)ppGpp were about the same length as the *relA* mutant or the *relA spoT* mutant (Fig. 4). These results are in general agreement with previous work in *E. coli* showing increased cell length in *relA spoT* mutants (86) and in *Pseudomonas aeruginosa* showing that strains lacking polyP become filamentous in stationary phase (87). The triple mutant *ppk relA spoT* contained filamentous cells as well (Fig. 4); however, these cells were much more heterogenous than any of the other mutants, appearing less stable with odd morphologies that we will explore in depth in the following sections. When grown on MOPS minimal media agarose pads, the mutant cells were no longer filamentous and had lengths more similar to the wild type (Fig. 4C) (compare, for example, the wild-type and *ppk relA* strains growing on MOPS in Videos SV1 and SV2). Nevertheless, even under these conditions, *relA*, *relA spoT*, and *ppk relA spoT* mutant cells were significantly longer than the wild type. The *ppk relA spoT* triple mutant immediately stopped growing on the MOPS agarose pad and slowly started to shrink over several hours (Video SV3). Over 3 hours on MOPS minimal media agarose pads, *ppk relA spoT* mutants shrank an average of 15.3% in total length (Video SV3; Table S1).

### Morphological defects of *ppk relA spoT* cells 2: displaced FtsZ ring formation and deviant cell division

**Fig 5:**
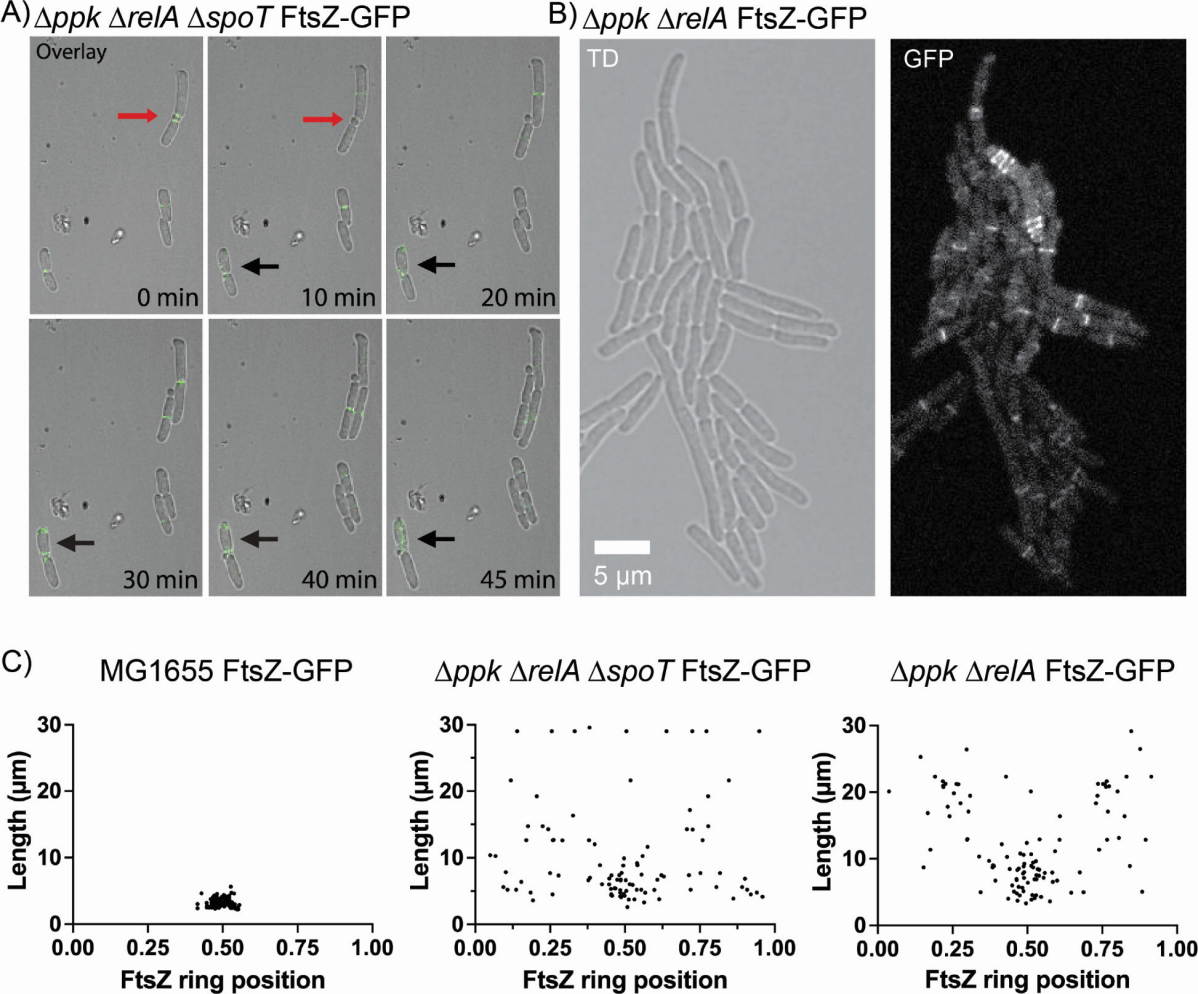
Strains lacking polyP and (p)ppGpp have disrupted cell division. (**A**) Confocal fluorescence time-lapse microscopy of the mutant *ppk relA spoT* FtsZ-GFP (MJG2405) on an LB agarose pad at 37°C. The triple mutant forms two Z-rings in the middle of the cell, releasing a mini-cell (red arrows). There are also two Z-rings that form at either pole of a single cell, both functional and releasing a mini cell (black arrows). Single channel images of TD and GFP alone can be viewed in Fig. S6. Examples of mini-cell formation and release can also be seen by transmission and cryo-electron microscopy (Fig. S9 and S11). (**B**) Confocal fluorescence time-lapse microscopy of the mutant *ppk relA* FtsZ-GFP (MJG2403) on an LB agarose pad at 37°C. This image shows a mutant forming three Z-rings at one pole, and at least three at the opposite pole as well, with no Z-rings forming in the middle of the cell, for a total of six Z-rings in a single cell. This cell continued to grow without lysing (Video SV5). (**C**) Quantification of FtsZ-ring location within the cell, and its relationship to cell length in *E. coli* strains MG1655 FtsZ-GFP (MJG2041), *ppk relA* FtsZ-GFP (MJG2403), and *ppk relA spoT* FtsZ-GFP (MJG2405). Cell length is shown on the y-axis, with the corresponding location of the FtsZ-ring on the x-axis as a fraction of the total cell length. Positions of FtsZ rings were obtained by measuring, in pixels, the distance between the center of the FtsZ band and the cell pole. Poles were arbitrarily designated either 0 or 1, with the midpoint of the total cell length being 0.5.

To determine where cell division was taking place in these filamentous cells, we used an FtsZ-GFP reporter to observe where the Z-ring was localizing within cells during cell division (88). During time-lapse microscopy, we observed the FtsZ reporter forming a Z-ring at the midpoint of wild-type cells as expected (Fig. S5; Video SV4) (89–91). The double *ppk relA* mutant and the triple mutant *ppk relA spoT* however showed that, while some FtsZ rings formed normally, there were many cells with profoundly disrupted Z-ring localization phenotypes. We observed cells containing multiple Z-rings within a single cell (Fig. 5A and B; Fig. S6), including instances with two Z-rings forming in the middle of a cell with both Z-rings constricting and resulting in the release of a non-growing mini-cell (Fig. 5A; Fig. S6).

Not only were multiple Z-rings forming at the midpoint of cells, but we also observed Z-rings forming at the poles of cells dividing and releasing mini-cells (Fig. 5A and B; Video SV5). FtsZ rings appeared to be able to form nearly anywhere along the length of the cell in the *ppk relA* double and *ppk relA spoT* triple mutant (Fig. 5C). In the *ppk relA* strain roughly 4.7% of cells observed had disrupted FtsZ ring placement, while the *ppk relA spoT* mutant had about 0.8% of cells with disrupted FtsZ ring placement (Table S1). We also observed one instance of an FtsZ ring forming at the side wall of a branching *ppk relA* cell, pinching off and releasing a mini-cell from the side wall of a branching cell (Fig. S7). Most cells did appear to divide normally when cell division occurred, with filamentous cells failing to localize a Z-ring until division occurred. The *ppk* and *relA spoT* mutants did not have these errors with FtsZ formation and formed Z-rings normally in LB at 37°C (Fig. S5).

**Fig 6:**
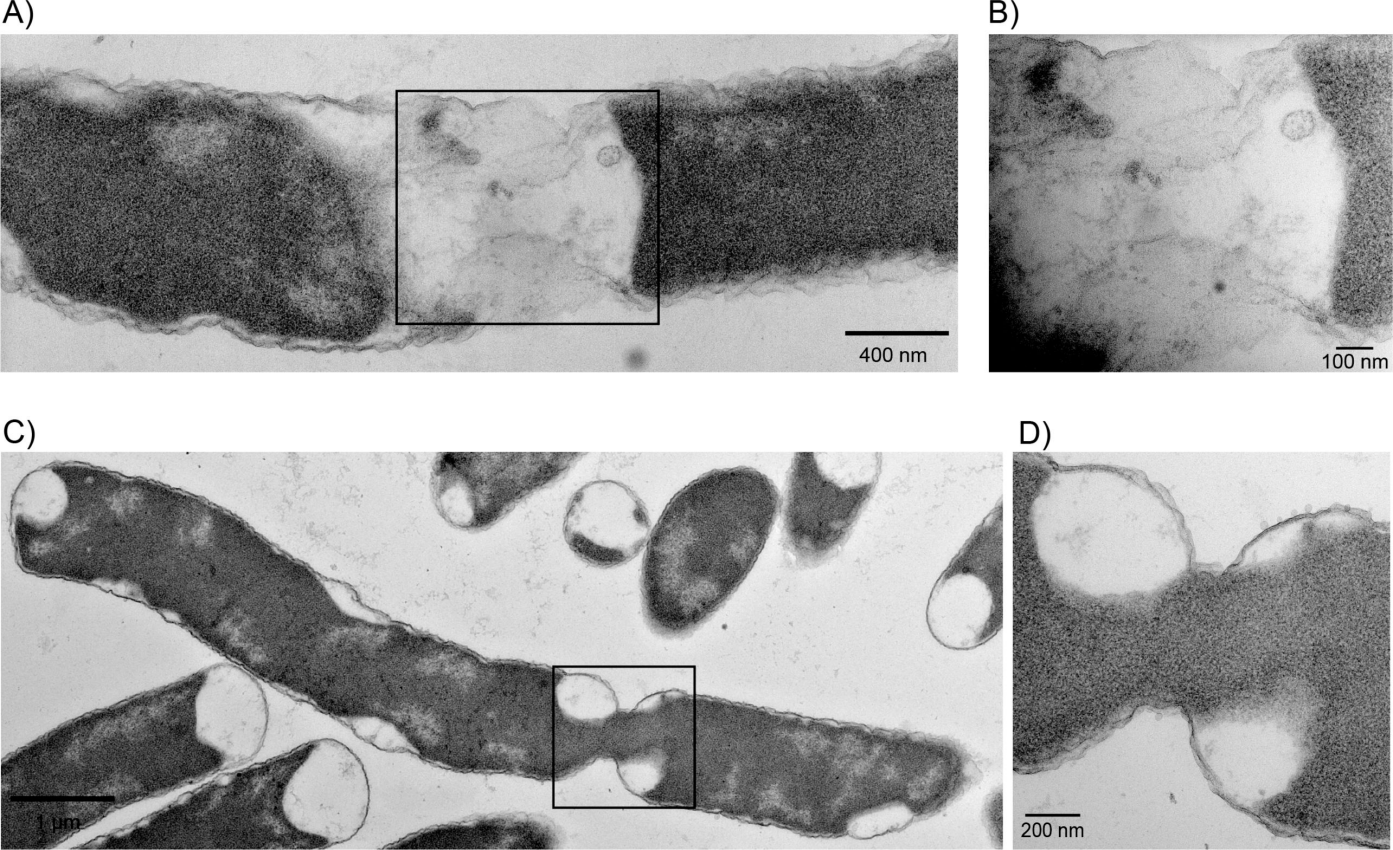
TEM of *ppk relA spoT* cells failing to divide properly. (**A**) TEM of *ppk relA spoT* (MJG1282) failing to divide properly. The cytoplasm of this cell appears to have condensed within the cell and away from the divisisome site but is still connected by what appears to be a small bridge of membrane that has yet to separate and could be the result of disrupted FtsZ-ring formation. (**B**) This image is the square section from (A) at a higher magnification. (**C**) TEM of *ppk relA spoT* (MJG1282) not completely forming a divisisome and staying connected through a bridge while still sharing cytoplasmic contents between the undivided cells. (**D**) This image is the square section from (C) at a higher magnification.

When the *ppk relA spoT* mutant was compared to the wild-type by transmission electron microscopy (TEM) and cryo-electron microscopy (CEM), we confirmed these observations (Fig. S8 to S11) and noticed other cell division defects in the triple mutant. We observed cells appearing to divide, but without forming a proper septum fully separating the dividing cells (Fig. 6). We observed a cell where cell division had mostly occurred and the cytoplasm was divided, with the two cells still connected by a membrane bridge (Fig. 6A and B). We also observed cells where cell division was actively occurring with a neck-like bridge between both cells still connecting the cytoplasm from one cell to another (Fig. 6C and D). None of these phenomena were observed in wild-type cells and could conceivably reflect a variety of defects in cell division in the *ppk relA spoT* mutant, either up- or downstream of the involvement of FtsZ itself.

### Morphological defects of *ppk relA spoT* cells 3: branched cells

**Fig 7:**
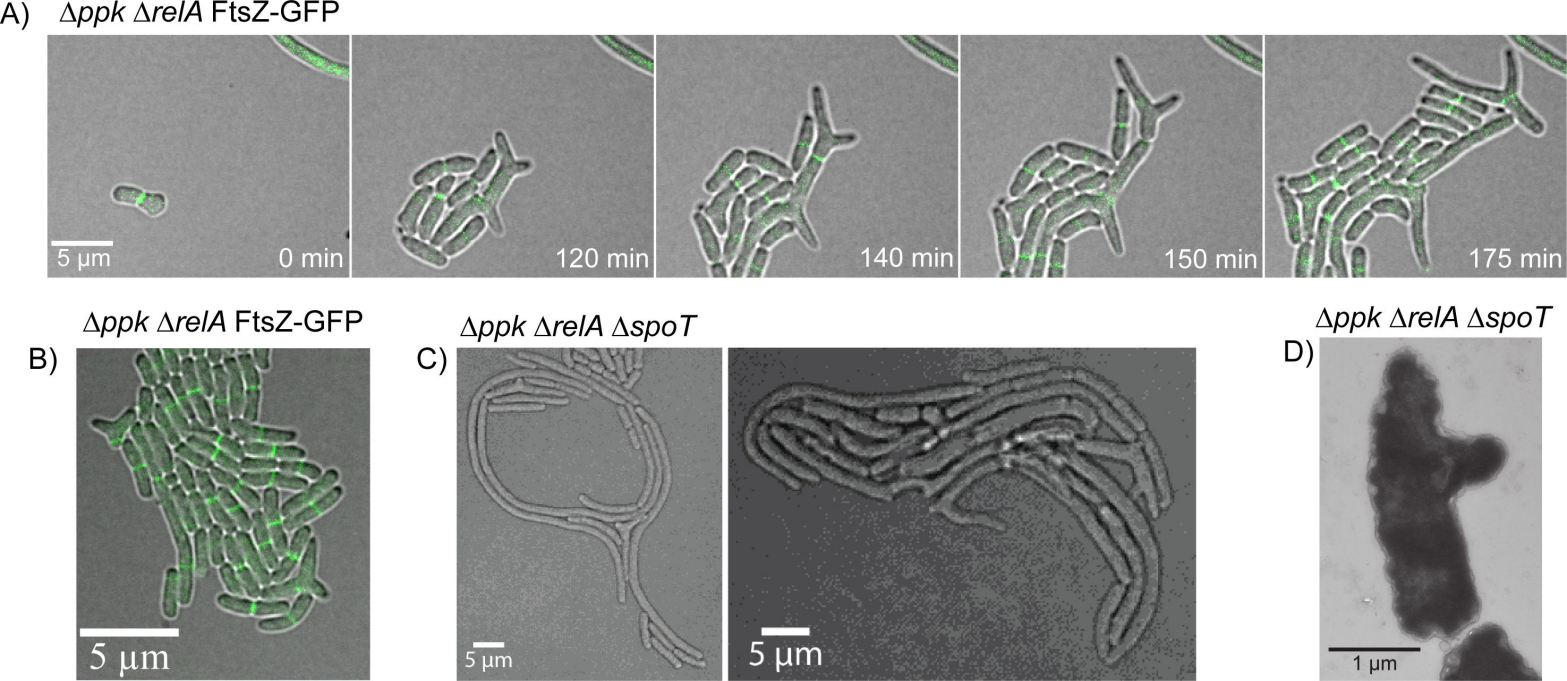
Cells lacking polyP and (p)ppGpp can develop branching cell morphologies. (**A**) Confocal fluorescence time-lapse microscopy of the mutant *ppk relA* FtsZ-GFP (MJG2403) on an LB agarose pad at 37°C showing branching cells. Branched cells are still capable of dividing and growing. (**B**) Still image of confocal fluorescent microscopy of *ppk relA* FtsZ-GFP mutant (MJG2403) mutant on LB agarose pad at 37°C showcasing branched cells. (**C**) Still image of confocal microscopy of *∆ppk ∆relA ∆spoT* (MJG1282) mutant on LB agarose pad at 37°C showcasing branched cells and other very odd cell morphologies. (**D**) Transmission electron microscopy image of *ppk relA spoT* (MJG1282) grown in LB at 37°C until log phase prior to imaging. This image shows a cell developing what appears to be a branching pole developed from the sidewall of the cell.

While observing the *ppk relA* double mutant and the *ppk relA spoT* triple mutants, we discovered that these mutants were able to form very unusual branched cells, along with other odd and unexpected cell morphologies. Only cells lacking both polyP and one or both (p)ppGpp synthetases were able to form branched cells, including instances of cells with more than three distinct poles (Fig. 7A through C; Fig. S7). In the *ppk relA* double mutant, we were able to observe a branched cell developing over time with FtsZ still capable of forming Z-rings and causing cell division (Fig. 7A). The frequency of cell branching was relatively low but contrasted starkly with the fact that it never occurred in wild-type, *ppk*, *relA*, or *relA spoT* strains. The double mutant *ppk relA* developed branched cells slightly less frequently (~1.9% of cells) than the triple mutant *ppk relA spoT* (~2.2% of cells) (Table S1), but there was less uniformity in branched cells of the triple mutant. The triple mutant developed into more heterogeneous branching cells with more noticeable defects in cell wall morphology including the formation of spheroplasts (Fig. 7C; Videos SV6 to SV8). The development of branched cells did not depend on the presence of the FtsZ-GFP reporter, as we observed the same morphologies in strains lacking the reporter (Fig. 7C). We also observed branching cells by TEM (Fig. 7D). These results may suggest a combined or redundant role for polyP and (p)ppGpp in regulating cell wall synthesis and/or integration of newly synthesized peptidoglycan, defects in which have been reported to result in branching in *E. coli* cells (92, 93).

### Morphological defects of *ppk relA spoT* cells 4: cell envelope defects and cytoplasmic condensation

**Fig 8:**
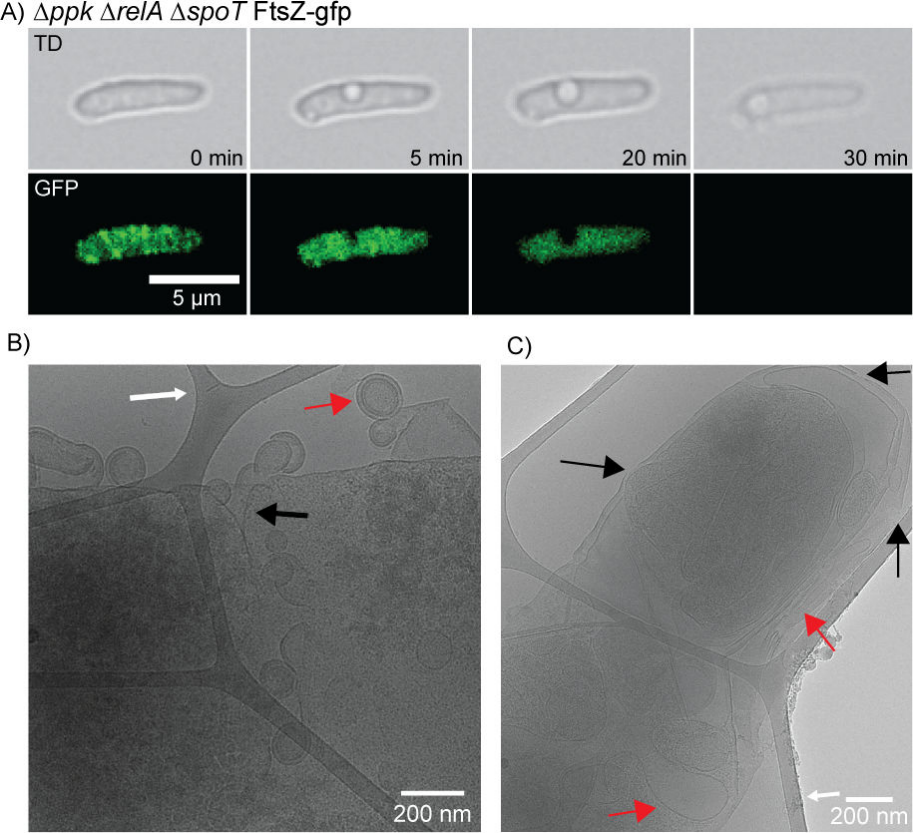
*ppk relA spoT* triple mutant cells can develop perforated membranes, leaking cytoplasmic contents out of the cell. (**A**) Time-lapse fluorescent microscopy of the *ppk relA spoT* mutant with the FtsZ-GFP reporter (MJG2405) showing empty space within the cell. FtsZ appears to fail to localize within the cell prior to losing its cytoplasmic contents just before cell death occurs. (**B**) Cryo-electron microscopy image of *ppk relA spoT* mutant (MJG2405) showing what appears to be a leaking cell wall with cytoplasmic contents blebbing off. White arrows point to the carbon lattice that cells were suspended on for imaging in CEM. There appears to be an invagination of the cell wall where cytoplasmic contents seem to be being released (black arrow). The cytoplasmic contents appear to be surrounded by a fully intact cell wall including an outer membrane, peptidoglycan, and inner membrane (red arrow). (**C**) CEM image of *ppk relA spoT* mutant (MJG2405) showing what appears to be holes in the cellular membrane (black arrows) where cytoplasmic contents and fluid may be lost. There also appears to be cytoplasm condensation, likely from fluid loss, with the cytoplasm condensing with the cell wall and membrane folding in on itself (red arrow).

Another unexpected phenotype we noted while observing the *ppk relA spoT* triple mutant was the presence of cells developing what appear as “holes” or void spaces within the bacterial cell, appearance of which preceded the leakage of cytoplasmic contents out of the cell, as represented by the FtsZ-GFP fusion protein (Fig. 8A). To investigate this phenotype more closely, we performed cryo-electron microscopy of the triple mutant, looking for evidence of disrupted cellular membranes. We imaged what appears to be an invagination of the cell wall, with cytoplasmic contents being released as blebs from the cell surrounded by a cell wall and membranes (Fig. 8B). The site where cytoplasmic contents appear to be “leaking” from may be a site of cell division, as we saw similar invaginated structures form in dividing cells (Fig. S10 and S11). We also observed disruptions in the outer membrane of the triple mutant (Fig. 8B), with what appears to be cytoplasmic contents condensing oddly within the cell (Fig. 8B; Fig S12 and S13).

**Fig 9:**
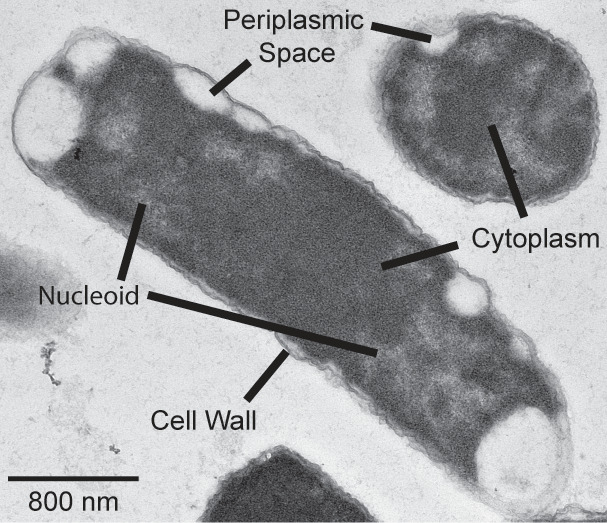
Transmission electron micrograph of *ppk relA spoT* mutant showing plasmolysis in both cross-sectional and trans-sectional viewpoint. TEM of *ppk relA spoT* (MJG1282) shows the inner membrane appearing to shrink and pull away from the cell wall (plasmolysis), leaving large periplasmic spaces within the cell. We can see here in both the longitudinal and horizontal axis of the cell wall surrounding the cell with large open spaces around the perimeter of the cell where there appears to be no cytoplasm creating these periplasmic spaces and resulting in plasmolysis. The cell appears to still be actively dividing as we can see in the longitudinal axis it appears that the nucleoid of the cell is being restricted to the poles of the cell away from the midline as we would expect in cells about to divide.

Using TEM, we observed *ppk relA spoT* mutant cells that appeared to have their cytoplasm and inner membrane shrunk away from the cell wall, creating large periplasmic spaces in ~1.1% of cells observed (Table S1), a phenomenon known as plasmolysis (Fig. 8 and 9; Fig. S14 and S15). Plasmolysis has been known to be caused by a hyperosmotic shock in bacteria, although in that case the cytoplasmic shrinkage is relatively uniform among cells in a population (94, 95) and as shown in Fig. S1A, changes in medium osmolarity do not impact the growth of the *ppk relA spoT* mutant. These results suggest there might be a mechanism by which polyP and (p)ppGpp co-regulate turgor pressure or potentially for dysregulation of efflux pumps causing the loss of cytoplasmic contents and subsequent efflux of water. This dehydration, however, could also be due to loss of water through a leaking membrane (Fig. 8) or other unknown mechanisms. In another paper reporting similar-appearing void spaces in bacterial cytoplasms, the authors believed they were seeing cytoplasmic condensation caused by disruptions in the cell envelope due to treatment with sublethal concentrations of antibiotics (96), in which case our observations might imply a role for polyP and (p)ppGpp in synthesis and/or incorporation of the newly synthesized peptidoglycan into the cell wall. Regardless of the underlying mechanism(s), these results indicate that in a *ppk relA spoT* mutant, some cells are unable to properly maintain their growing cell wall or membranes.

### Stringent alleles of RNA polymerase restore some, but not all phenotypes of a *ppk relA spoT* mutant

**Fig 10:**
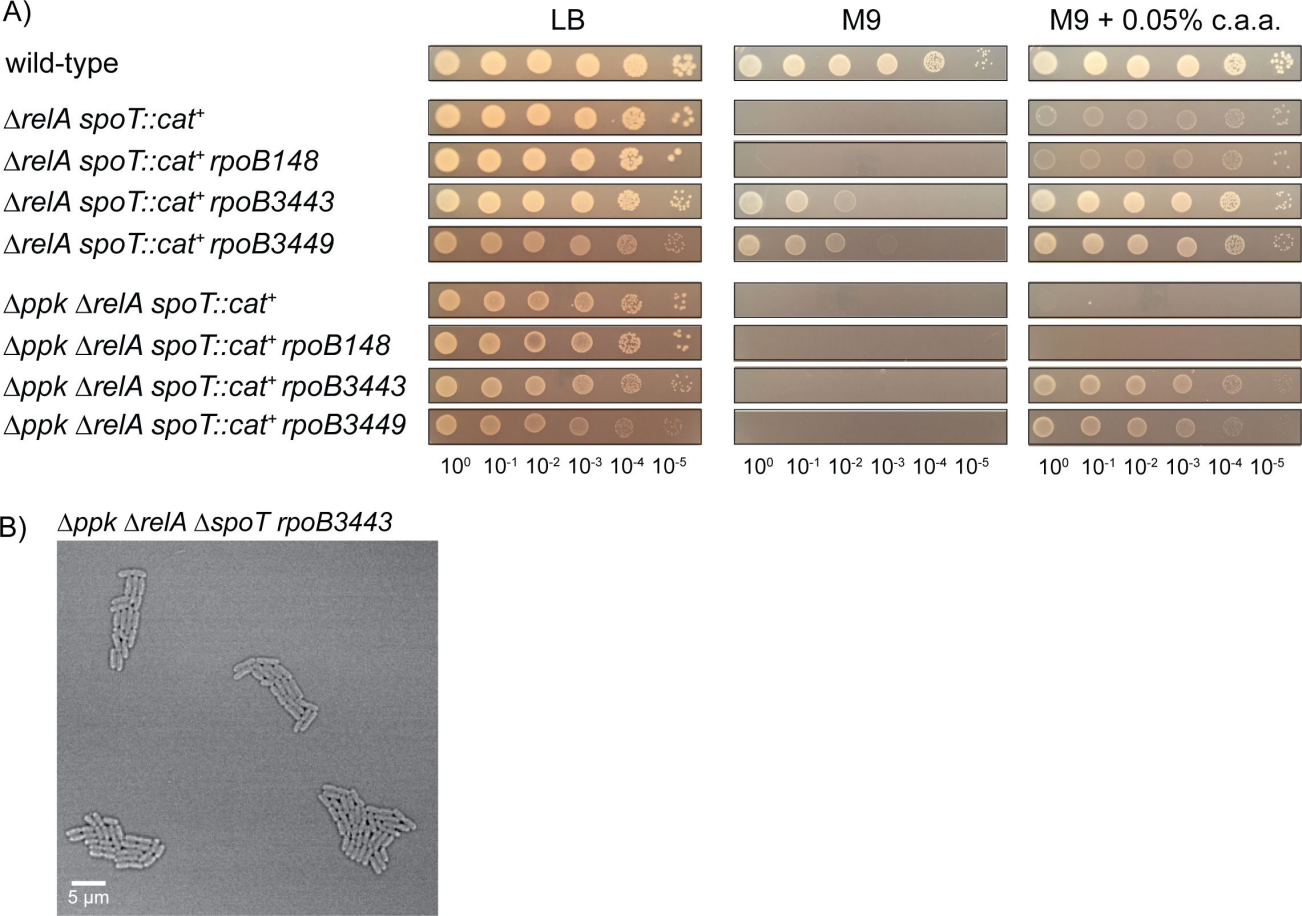
Stringent alleles of RNA polymerase restore growth of *ppk relA spoT* mutants on minimal medium with casamino acids and rescue morphological defects on rich medium. (**A**) *E. coli* strains MG1655 (wild-type), MJG1136 (MG1655 ∆*relA782 spoT207::cat*^+^), MJG1137 (MG1655 ∆*ppk-749* ∆*relA782 spoT207::cat*^+^), MJG1237 (MG1655 ∆*relA782 spoT207::cat*^+^ *rpoB3449*), MJG1241 (MG1655 ∆*ppk-749* ∆*relA782 spoT207::cat*^+^ *rpoB3449*), MJG1579 (MG1655 ∆*relA782 spoT207::cat*^+^ *rpoB3443*), MJG1580 (MG1655 ∆*relA782 spoT207::cat*^+^ *rpoB148*), MJG1581 (MG1655 ∆*ppk-749* ∆*relA782 spoT207::cat*^+^ *rpoB3443*), and MJG1582 (MG1655 ∆*ppk-749* ∆*relA782 spoT207::cat*^+^ *rpoB148*) were grown overnight in LB broth, then rinsed and normalized to an A_600_ = 1 in PBS. Aliquots (5 µL) of serially diluted suspensions were spotted on LB, M9 glucose, or M9 glucose containing 0.05% (w/v) casamino acids (c.a.a.) plates and incubated overnight at 37°C (representative image from at least three independent experiments). (**B**) Confocal microscopy of *ppk relA spoT rpoB3443* (MJG1581) grown on a LB agarose pad at 37°C.

The amino acid auxotrophy of *relA spoT* mutants can be rescued by mutations in RNA polymerase called stringent alleles, which mimic the regulatory effect of (p)ppGpp binding to RNA polymerase on transcription, including the activation of expression of amino acid synthesis operons (69, 70, 75, 76). Many of these mutations in RNA polymerase also confer rifampicin resistance (97). The stringent alleles *rpoB3443* and *rpoB3449* (70) were, as expected, able to restore the growth of a *relA spoT* mutant in the absence of casamino acids, while the rifampicin-resistant but non-stringent allele *rpoB148* (97) did not (Fig. 10A). Notably, *rpoB3443* and *rpoB3449* restored growth of the *ppk relA spoT* triple mutant in the presence of casamino acids, but not in their absence. Microscopic observation of a *ppk relA spoT rpoB3443* mutant grown on LB, however, revealed a dramatic restoration of wild-type cell morphology (Fig. 10B; Video SV9). The growth rates of the *ppk relA spoT* and *ppk relA spoT rpoB3443* mutants in LB, while considerably slower than the wild type, were not different from each other (Fig. S16), so this morphological rescue was not due to a change in growth rate.

These results taken together suggest that the nutritional (Fig. 1 to 3 and 10A) and morphological (Fig. 4 to 9 and 10B) phenotypes of the *ppk relA spoT* mutant can be genetically separated and that the morphological phenotypes of this strain in particular appear to be linked to transcriptional regulation by (p)ppGpp. By contrast, the combinatorial growth defect of the triple mutant on a minimal medium may be dependent on (p)ppGpp’s impacts on a protein or proteins other than RNA polymerase (66, 75, 76). These results also reinforce our conclusion that whatever compound(s) are present in LB that allow growth of the triple mutant (Fig. 3) are not likely to be amino acids.

## DISCUSSION

Connections between (p)ppGpp and polyP in *E. coli* have been suspected for decades (44, 62), but the nature and consequences of those connections have remained obscure (42, 43, 47, 98). We have now identified a striking combinatorial phenotype that clearly demonstrates that these two conserved “stress response” molecules play important linked roles in controlling fundamental metabolic processes under non-stress growth conditions. The fact that these phenotypes appear only when both (p)ppGpp and polyP are eliminated suggests that either one alone is sufficient to maintain more or less normal cells, and that therefore some critical pathway or pathways must be regulated by both molecules. The challenge that remains is to dissect the mechanism(s) by which (p)ppGpp and polyP coordinate these processes.

Both (p)ppGpp and polyP can act at multiple regulatory levels in the bacterial cell. The regulatory consequences of (p)ppGpp synthesis are better studied and include dramatic changes in the genome-wide transcriptome of *E. coli* due to (p)ppGpp binding to RNA polymerase and the transcription factor DksA (2, 63). (p)ppGpp can also direct regulation of a variety of other enzymes (1, 26, 66), including notably the exopolyphosphatase PPX, which is inhibited by (p)ppGpp (44, 55, 62). We do not know at this time whether PPX impacts the phenotypes reported here. More than 700 genes are transcriptionally regulated by (p)ppGpp, approximately 400 of which are inhibited and 300 are stimulated (including, to a modest extent, *ppk*)(99), but the exact list depends on growth conditions and on how (p)ppGpp synthesis is induced (99, 100). Although stringent alleles of RNA polymerase have been known for many decades and are thought to mimic the effects of (p)ppGpp binding (69, 70, 75, 76), to our knowledge no genome-wide characterization of their impact on transcription has been performed. More than 50 other proteins that bind (p)ppGpp in *E. coli* have been identified (66), and the impact of that binding has been characterized for only a subset of those proteins (1, 26, 66). No experiments have been reported to date examining the polyP protein interactome in *E. coli*. It also remains possible that some effects of PPK might be either indirect, for example by impacts on nucleotide pools (Fig. S2)(41) or entirely independent of polyP synthesis, although there are no consistently identified PPK protein-binding partners in the literature for protein-protein interactions in *E. coli* MG1655 (101–104).

PolyP can also act at multiple levels, although its impact is considerably less well characterized. PolyP interacts directly with the Lon protease, for example, to modulate its activity and substrate specificity (105–108), which could affect a very broad range of potential protein targets in the cell both directly and indirectly. In combination with Hfq, polyP is also able to silence transcription of some genes (109), and a ∆*ppk* mutant has substantial changes in its transcriptome and proteome (110), although how much of this is due to direct regulatory impacts as opposed to indirect responses to a lack of polyP remains unclear. Whether other *E. coli* proteins might be impacted by polyP binding is not well known, since to our knowledge no systematic study identifying polyP-binding proteins in bacteria has been performed (111, 112). The fact that we can genetically distinguish between the metabolic and morphological phenotypes of *ppk relA spoT* mutants (Fig. 10) strongly suggests that at least two different pathways are co-regulated by (p)ppGpp and polyP, which further complicates the problem.

Based on the existing literature, we can identify a few potential overlaps between the (p)ppGpp and polyP regulons in *E. coli* which might be relevant to the phenotypes we observe in *ppk relA spoT* mutants. As one example, (p)ppGpp binds to the DapB protein (66) and upregulates transcription of many of the *dap* genes involved in the synthesis of the peptidoglycan precursor diaminopimelate (99, 113), while in a ∆*ppk* mutant, Varas et al. (110) reported an increase in DapA protein levels and a decrease in *dapF* transcription. In another example, FtsY, an essential component of the signal recognition particle that delivers integral membrane proteins to the inner membrane (114), is allosterically inhibited by (p)ppGpp binding (66, 115), modestly downregulated transcriptionally by (p)ppGpp (99), and its transcription is increased in a ∆*ppk* mutant (110). Either of these pathways could conceivably contribute to the cell envelope defects we observe in *ppk relA spoT* mutants (Fig. 7 to 9), but since each of those experiments was performed under different conditions and in varying strain backgrounds it is not possible to make definite conclusions about whether these potential points of regulatory overlap are either real or meaningful, and certainly not about whether they contribute to the phenotypes of *ppk relA spoT* strains. We are currently working to characterize the transcriptional and post-transcriptional impacts of (p)ppGpp and polyP on *E. coli* under growth conditions where we observe *ppk relA spoT* phenotypes, with the goal of systematically identifying genes, proteins, and pathways impacted by both molecules.

Perhaps the most surprising and most difficult to explain of the phenotypes of the *ppk relA spoT* mutant is the mislocalization of Z-rings (Fig. 5 to 7). The positioning of FtsZ at the center of the cell is a highly regulated part of cell division (116). *E. coli* has two major systems for preventing formation of Z-rings anywhere other than mid-cell: the MinCDE system that inhibits Z-ring formation at the poles (117) and the SlmA nucleoid exclusion protein that prevents Z-ring formation around the DNA nucleoid (118). Neither of these systems has been reported to be impacted by either polyP or (p)ppGpp, although examination of published transcriptome data indicates that *minD* and *minE* are slightly upregulated and *slmA* is slightly downregulated by (p)ppGpp (99). Mutants lacking *minCDE* have Z-ring distributions (119) reminiscent of those we observed in *ppk relA* and *ppk relA spoT* mutants (Fig. 5C), but have not been reported to have any of the other growth or morphological defects we observed. Overexpression of FtsZ can also result in production of multiple Z-rings within cells, including both mid-cell and polar localizations (120), reminiscent of some of our observations of *ppk relA spoT* mutants containing many concurrent Z-rings (Fig. 5). FtsZ regulation is complex, but not known to depend directly on either (p)ppGpp or polyP (116). Our data strongly suggest that there are important unknowns remaining in our understanding of the regulation of proper cell division in *E. coli*.

In *E. coli*, both (p)ppGpp and polyP are present at very low levels under non-stress conditions and those levels are strongly increased by various stresses, which is critical for the ability of the bacteria to survive those stress treatments (2–4). However, the cell morphology phenotypes we report here for the *ppk relA spoT* mutant are in a rich LB medium, meaning that those phenotypes depend on unstimulated basal levels of both (p)ppGpp and polyP. Levels of polyP are extremely low in *E. coli* grown in LB, often below the limit of detection for standard assays (Fig. S3B) (41, 43, 47, 77, 121). There is an increasing appreciation in the literature that basal levels of (p)ppGpp make important contributions to diverse aspects of *E. coli* physiology (122), including cell division (123). The same seems likely to be true of basal polyP levels since substantial changes in gene expression and proteome composition are seen in ∆*ppk* mutants grown in rich medium (109, 110), but more sensitive detection methods may need to be developed to probe this in more detail (124).

A final point worth considering is the surprising heterogeneity of the *ppk relA spoT* cells. As shown in Fig. 4 to 9; Table S1, not every cell of the triple mutant has the same morphological defects. Some of them look fairly normal, some are filamentous, some have mislocalized Z-rings, and a few develop more severe defects, including branching, spheroplast formation, void spaces, or lysis. What underlies this diversity and what distinguishes individual cells that do well from cells that do not is an intriguing unanswered question.

## MATERIALS AND METHODS

### Databases and primer design

We obtained gene and protein sequences and other information from the Integrated Microbial Genomes database (125) and from the EcoCyc (113) and designed PCR and sequencing primers with Web Primer (www.candidagenome.org/cgi-bin/compute/web-primer). Mutagenic primers were designed with PrimerX (www.bioinformatics.org/primerx/index.htm).

### Bacterial strains and growth conditions

All strains and plasmids used in this study are listed in Table 1. We grew *E. coli* at 37°C in lysogeny broth (LB)(126), containing 5 g L^−1^ NaCl unless otherwise indicated, in M9 minimal medium (78) containing 4 g L^−1^ glucose, or in MOPS minimal medium (79) containing 2 or 4 g L^−1^ glucose. Solid media contained 1.5% (wt/vol) agar (Becton Dickinson cat. #214010). Ampicillin (100 µg mL^−1^), chloramphenicol (17.5 or 35 µg mL^−1^), kanamycin (25 or 50 µg mL^−1^), or rifampicin (50 µg mL^−1^) were added when appropriate. Nutritional supplements used were yeast synthetic dropout mix supplement (Sigma Aldrich cat. #Y1501), casamino acids (Fisher Scientific cat. #BP1424), tryptone (Fisher Scientific cat. #BP1421), or yeast extract (Becton Dickinson cat. #288620). For growth curves, *E. coli* strains of interest were grown overnight at 37°C with shaking in LB, then normalized to A_600_ = 1 and rinsed three times with sterile PBS. The resulting cell suspensions were diluted 1:40 into fresh media. Growth curves were performed in clear 96-well plates in Tecan Spark or Sunrise plate readers, incubating at 37°C with shaking and measuring A_600_ at 30 minutes of intervals for 24 hours.

**TABLE 1:** Strains and plasmids used in this study^*a*^

Strain or plasmid	Marker(s)	Relevant genotype	Source
*E. coli* strains			
MG1655		F^−^, λ^−^, *rph-1 ilvG^−^ rfb-50*	(127)
CF1693(M+)	Cm^R^ Kn^R^	MG1655 λ^+^ϕ80^+^ *relA251*::*kan^+^ spoT207*::*cat^+^ rpoB1693* (encoding RpoB^N1129K^) *rpoD1693* (encoding RpoD^D64G^)	(128, 129)
BH330	Ap^R^	MG1655 λ*attB::P_lac_-gfp-ftsZ*, *bla*^+^	(88)
MJG0224		MG1655 ∆*ppk-749*	(41)
MJG0226		MG1655 ∆*relA782*	(43)
MJG0344		MG1655 ∆*rpoS746*	(43)
MJG1090	Kn^R^	MG1655 ∆*ppk-749* ∆*relA782*::*kan*^+^	
MJG1097	Kn^R^	MG1655 ∆*rpoS746* ∆*relA782*::*kan*^+^	
MJG1116		MG1655 ∆*ppk-749* ∆*relA782*	
MJG1119		MG1655 ∆*rpoS746* ∆*relA782*	
MJG1136	Cm^R^	MG1655 ∆*relA782 spoT207*::*cat*^+^	(43)
MJG1137	Cm^R^	MG1655 ∆*ppk-749* ∆*relA782 spoT207*::*cat*^+^	
MJG1236	Rif^R^	MG1655 ∆*ppk-749* ∆*relA782 rpoB3449* (encoding RpoB^∆Ala532^)	
MJG1237	Cm^R^ Rif^R^	MG1655 ∆*relA782 spoT207*::*cat*^+^ *rpoB3449* (encoding RpoB^∆Ala532^)	(43)
MJG1241	Cm^R^ Rif^R^	MG1655 ∆*ppk-749* ∆*relA782 spoT207*::*cat*^+^ *rpoB3449* (encoding RpoB^∆Ala532^)	
MJG1282	Kn^R^	MG1655 ∆*ppk-749* ∆*relA782* ∆*spoT1000*::*kan*^+^	
MJG1287	Kn^R^	MG1655 ∆*relA782* ∆*spoT1000*::*kan*^+^	(43)
MJG1575	Rif^R^	MG1655 ∆*relA782 rpoB3443* (encoding RpoB^L533P^)	
MJG1576	Rif^R^	MG1655 ∆*relA782 rpoB148* (encoding RpoB^D517V^)	
MJG1577	Rif^R^	MG1655 ∆*ppk-749* ∆*relA782 rpoB3443* (encoding RpoB^L533P^)	
MJG1578	Rif^R^	MG1655 ∆*ppk-749* ∆*relA782 rpoB148* (encoding RpoB^D517V^)	
MJG1579	Cm^R^ Rif^R^	MG1655 ∆*relA782 spoT207*::*cat*^+^ *rpoB3443* (encoding RpoB^L533P^)	
MJG1580	Cm^R^ Rif^R^	MG1655 ∆*relA782 spoT207*::*cat*^+^ *rpoB148* (encoding RpoB^D517V^)	
MJG1581	Cm^R^ Rif^R^	MG1655 ∆*ppk-749* ∆*relA782 spoT207*::*cat*^+^ *rpoB3443* (encoding RpoB^L533P^)	
MJG1582	Cm^R^ Rif^R^	MG1655 ∆*ppk-749* ∆*relA782 spoT207*::*cat*^+^ *rpoB148* (encoding RpoB^D517V^)	
MJG2401	Ap^R^	MG1655 λ*attB::P_lac_-gfp-ftsZ*, *bla*^+^	
MJG2402	Ap^R^	MG1655 ∆*ppk-749* λ*attB::P_lac_-gfp-ftsZ*, *bla*^+^	
MJG2403	Kn^R^ Ap^R^	MG1655 ∆*ppk-749* ∆*relA782*::*kan*^+^ λ*attB::P_lac_-gfp-ftsZ*, *bla*^+^	
MJG2404	Kn^R^ Ap^R^	MG1655 ∆*relA782* ∆*spoT1000*::*kan*^+^ λ*attB::P_lac_-gfp-ftsZ*, *bla*^+^	
MJG2405	Kn^R^ Ap^R^	MG1655 ∆*ppk-749* ∆*relA782* ∆*spoT1000*::*kan*^+^ λ*attB::P_lac_-gfp-ftsZ*, *bla*^+^	
Plasmids			
pUC18	Ap^R^	*bla* ^+^	(130)
pKD46	Ap^R^	λ Red*^+^, bla^+^*	(131)
pKD4	Kn^R^	*kan* ^+^	(131)
pCP20	Ap^R^ Cm^R^	Flp^+^ *bla*^+^*cat*^+^	(131)
pPPK1	Cm^R^	*ppk*^+^ *cat*^+^	(41)
pPPK8	Ap^R^	*ppk* ^+^ *bla* ^+^	
pSPOT1	Ap^R^	*spoT* ^+^ *bla* ^+^	
pSPOT2	Ap^R^	*spoT*^G217A, C219T^ (encoding SpoT^D73N^) *bla*^+^	
pSPOT3	Ap^R^	*spoT*^G775A, C777T^ (encoding SpoT^D259N^) *bla*^+^	
pSPOT4	Ap^R^	*spoT*^G217A, C219T, G775A, C777T^ (encoding SpoT^D73N, D259N^) *bla*^+^	
pPPK30	Ap^R^	*ppk*^G733A^ (encoding PPK^E245K^) *bla*^+^	(42)

^*a*^ Unless otherwise indicated, strains and plasmids were generated in the course of this work. Abbreviations: Ap^R^, ampicillin resistance; Cm^R^, chloramphenicol resistance; Kn^R^, kanamycin resistance; Rif^R^, rifampicin resistance.

### Strain construction

All *E. coli* strains used in this study were derivatives of wild-type strain MG1655 (F^-^, λ^-^, *rph-1 ilvG^-^ rfb-50*) (127). We confirmed chromosomal mutations by PCR and whole-genome sequencing (SeqCenter, Philadelphia, PA). All strains derived from CF1693(M+) (128, 129) were confirmed free of contamination with λ and ϕ80 phage by PCR (43).

We used P1*vir* phage transduction (43, 132) to move the ∆*relA782::kan*^+^ allele from the Keio collection (133) into strains MJG0224 (∆*ppk-749*) (41) and MJG0344 (∆*rpoS746*) (43) to generate strains MJG1090 (∆*ppk-749* ∆*relA782::kan*^+^) and MJG1097 (∆*rpoS746* ∆*relA782::kan*^+^). We then used plasmid pCP20 (131) to resolve the kanamycin resistance cassettes in those strains, generating strains MJG1116 (∆*ppk-749* ∆*relA782*) and MJG1119 (∆*rpoS746* ∆*relA782*). We used P1*vir* phage transduction to move the *spoT207::cat*^+^ allele from CF1693(M+) (128, 129) into MJG1116 (∆*ppk-749* ∆*relA782*), generating strain MJG1137 (∆*ppk-749* ∆*relA782 spoT207::cat*^+^). The *spoT* gene of strain MJG1116 (∆*ppk-749* ∆*relA782*) was replaced with a pKD4-derived kanamycin resistance cassette by recombineering (131) using primers 5′ GTT ACC GCT ATT GCT GAA GGT CGT CGT TAA TCA CAA AGC GGG TCG CCC TTG GTG TAG GCT GGA GCT GCT TC 3′ and 5′ GGC GAG CAT TTC GCA GAT GCG TGC ATA ACG TGT TGG GTT CAT AAA ACA TTA CAT ATG AAT ATC CTC CTT AG 3′, yielding strain MJG1282 (∆*ppk-749* ∆*relA782 spoT1000::kan*^+^).

Oligo-directed recombineering (134) was used to construct chromosomal *rpoB3449*, *rpoB3443*, and *rpoB148* alleles (43, 70, 97, 135–137) using the mutagenic primers 5′ CCT GCA CGT TCA CGG GTC AGA CCG CCT GGA CCA AGA GAA ATA CGA CGT TTG TGC GTA ATC TCA GAC AGC G 3′, 5′ AAG CCT GCA CGT TCA CGG GTC AGA CCG CCG GGA CCG GGG GCA GAG ATA CGA CGT TTG TGC GTA ATC TCA GAC A 3′, and 5′ ATA CGA CGT TTG TGC GTA ATC TCA GAC AGC GGA TTA TTT TGG ACC ATA AAC TGA GAC AGC TGG CTG GAA CCG A 3′, respectively, each of which contained four 5′ phosphorothiorate linkages to stabilize the primers. The *rpoB3449* primer deletes nucleotides G1593 through A1596 of *rpoB*, removing the codon for alanine 532 of RpoB, and also incorporates silent mutations in four adjacent codons (C1590T, C1593T, C1599T, and C1602T) to avoid mismatch repair (43). The *rpoB3443* primer mutates nucleotide T1598 of *rpoB* to C, changing leucine 533 to proline, and incorporates silent mutations in four adjacent codons (C1593T, A1596C, C1602T, and A1605C). The *rpoB148* primer mutates nucleotide A1547 of *rpoB* to T, changing aspartic acid 516 to valine, and incorporates silent mutations in three adjacent codons (G1551A, C1554T, and C1557T). Strains MJG0226 (∆*relA782*) or MJG1116 (∆*ppk-749* ∆*relA782*) were transformed with pKD46 (131), induced to express the λ Red recombinase, and electroporated with 250 pmol of mutagenic primer. Recombinant colonies were selected at 37°C on LB plates containing rifampicin. The sequence of *rpoB* alleles was confirmed by PCR amplification of a fragment of *rpoB* with primers 5′ GAT GTT ATG AAA AAG CTC 3′ and 5′ CTG GGT GGA TAC GTC CAT 3′ and Sanger sequencing of the resulting product (UAB Heflin Sequencing Core Facility). After curing pKD46 by growth at 37°C, this yielded strains MJG1236 (∆*ppk-749* ∆*relA782 rpoB3449*), MJG1575 (∆*relA782 rpoB3443*), MJG1576 (∆*relA782 rpoB148*), MJG1577 (∆*ppk-749* ∆*relA782 rpoB3443*), and MJG1578 (∆*ppk-749* ∆*relA782 rpoB148*).

We used P1*vir* phage transduction to move the *spoT207::cat*^+^ allele from CF1693(M+) (128, 129) into MJG1236 (∆*ppk-749* ∆*relA782 rpoB3449*), generating strain MJG1237 (∆*ppk-749* ∆*relA782 spoT207::cat*^+^*rpoB3449*). We used P1*vir* phage transduction to move the *spoT207::cat*^+^ allele from MJG1136 (∆*relA782 spoT207::cat*^+^) (43) into strains MJG1575 (∆*relA782 rpoB3443*), MJG1576 (∆*relA782 rpoB148*), MJG1577 (∆*ppk-749* ∆*relA782 rpoB3443*), and MJG1578 (∆*ppk-749* ∆*relA782 rpoB148*), yielding strains MJG1579 (∆*relA782 spoT207::cat*^+^ *rpoB3443*), MJG1580 (*relA782 spoT207::cat*^+^ *rpoB148*), MJG1581 (∆*ppk-749* ∆*relA782 spoT207::cat*^+^ *rpoB3443*), and MJG1582 (∆*ppk-749* ∆*relA782 spoT207::cat*^+^ *rpoB148*) respectively.

To construct fluorescent reporter strains, we used P1*vir* phage transduction to move the λ*attB::P_lac_-gfp-ftsZ* allele from strain BH330 (88) into strains MG1655, MJG0224 (∆*ppk-749*), MJG1090 (∆*ppk-749* ∆*relA782::kan*^+^), MJG1287 (∆*relA782* ∆*spoT1000::kan*^+^), or MJG1282 (∆*ppk-749* ∆*relA782* ∆*spoT1000::kan*^+^), selecting for the linked ampicillin resistance marker. This resulted in strains MJG2401 (λ*attB::P_lac_-gfp-ftsZ*, *bla*^+^), MJG2402 (∆*ppk-749* λ*attB::P_lac_-gfp-ftsZ*, *bla*^+^), MJG2403 (∆*ppk-749* ∆*relA782::kan*^+^ λ*attB::P_lac_-gfp-ftsZ*, *bla*^+^), MJG2404 (∆*relA782* ∆*spoT1000::kan*^+^ λ*attB::P_lac_-gfp-ftsZ*, *bla*^+^), and MJG2405 (∆*ppk-749* ∆*relA782* ∆*spoT1000::kan*^+^ λ*attB::P_lac_-gfp-ftsZ*, *bla*^+^).

To construct strains expressing the hyperactive PPK* allele PPK^E245K^ (*ppk*^G733A^)(85), we electroporated pPPK30 (*ppk*^G733A^*bla*^+^) (42) into strains MG1655, MJG0224 (∆*ppk-749*), MJG1116 (∆*ppk* ∆*relA*)*,* MJG1287 (∆*relA782* ∆*spoT1000::kan*^+^), or MJG1282 (∆*ppk-749* ∆*relA782* ∆*spoT1000::kan*^+^). This resulted in MJG2577 (MG1655 pPPK30 (*ppk*^G733A^*bla*^+^)), MJG2578 (∆*ppk-749* pPPK30 (*ppk*^G733A^*bla*^+^)), MJG2579 (∆*relA* pPPK30 (*ppk*^G733A^*bla*^+^)), MJG2580 (∆*ppk-749* ∆*relA782::kan*^+^ pPPK30 (*ppk*^G733A^*bla*^+^)), MJG2581 (∆*relA782* ∆*spoT1000::kan*^+^ pPPK30 (*ppk*^G733A^*bla*^+^)), and MJG2582 (∆*ppk-749* ∆*relA782* ∆*spoT1000::kan*^+^ pPPK30 (*ppk*^G733A^*bla*^+^)).

### Plasmid construction

The *E. coli* MG1655 *ppk* coding sequence (2,067 bp) plus 20 bp of the upstream sequence was subcloned from pPPK1 (41) into the *Kpn*I and *Hin*dIII sites of plasmid pUC18 (130) to generate plasmid pPPK8 (*ppk*^+^ *bla*^+^). The *spoT* coding sequence (2,109 bp) was amplified from *E. coli* MG1655 genomic DNA with primers 5′ AGA TCT AGA TTG TAT CTG TTT GAA AGC CTG AAT C 3′ and 5′ CTT AAG CTT TTA ATT TCG GTT TCG GGT GAC 3′ and cloned into the *Xba*I and *Hin*dIII sites of plasmid pUC18 (130) to generate plasmid pSPOT1 (*spoT*^+^ *bla*^+^). We used single primer site-directed mutagenesis (138) to mutate pSPOT1 with primers 5′ GGC GGC GCT GCT GCA TAA TGT GAT TGA AGA TAC TCC 3′ and 5′ CGT TTT CAC TCG ATC ATG AAT ATC TAC GCT TTC CGC GTG 3′. This yielded pSPOT2, containing a *spoT*^G217A,C219T^ allele (encoding SpoT^D73N^), and pSPOT3, containing a *spoT*^G775A,C777T^ allele (encoding SpoT^D259N^). We then used single primer site-directed mutagenesis (138) to further mutate pSPOT3 with primer 5′ GGC GGC GCT GCT GCA TAA TGT GAT TGA AGA TAC TCC 3′. This yielded pSPOT4, containing a *spoT*^G217A,C219T,G775A,C777T^ allele (encoding SpoT^D73N, D259N^)

### (p)ppGpp Quantification

Cultures were grown overnight in LB, then subcultured into 5 mL of LB for each sample, and grown with shaking at 37°C for 2–3 hours until cells started to grow exponentially (OD_600_ = ~0.1). Cells were put into two different 50 mL conical tubes containing 2 mL of the strain of interest, rinsed three times with sterile PBS, and resuspended in MOPS glucose medium. M9 media cannot be used for ^32^P quantification as the free phosphate within the M9 will also exchange for the radiolabeled phosphate, and thus MOPS was used for all (p)ppGpp assays. To one tube, we added ^32^P (Phosphorus-32 Radionuclide, 1mCi (37 MBq) (Revvity) to a final concentration of 20 µCi/mL (139). Cultures were incubated with shaking at 37°C until they reached an OD_600_ between 0.4 and 0.5 (for strains able to replicate in MOPS medium), then 200 µL culture was added to 40 µL of 2 M formic acid (140), incubated on ice for 15–60 min, then centrifuged at 16,000× *g* for 2 minutes at 4°C and supernatants were stored at −20°C. Thin-layer chromatography (TLC) was carried out to visualize and quantify phosphorylated guanine nucleotides as previously described (140, 141). TLC plates were analyzed on a Typhoon Biomolecular Imager (Cytiva). Spot intensity was quantified using ImageQuant, and the (p)ppGpp quotient was expressed as a fraction of the total guanosine pool, that is (pppGpp +ppGpp)/(pppGpp +ppGpp + GTP + GDP) (139, 141, 142).

### Polyphosphate quantification

PolyP was extracted from overnight cultures of bacterial strains using EconoSpin silica spin columns (Epoch Life Sciences) as previously described (85), eluting samples in 200 µL of 25 mM HEPES (pH 8). PolyP was then quantified using the polyP-specific fluorescence dye JC-D7 (143, 144) by comparison to a standard curve of commercial polyP (Acros Organics) and normalized to total cellular protein (as determined by Bradford assay of cell lysates).

### Size fractionation of yeast extract

We dissolved yeast extract in water (0.25 g mL^−1^) to make a concentrated stock solution. We placed the resulting solution in a 3,500 Da MWCO Slide-A-Lyzer cassette (ThermoFisher) and dialyzed it against distilled water to remove components smaller than 3,500 Da. Similarly, we used a 3,000 Da MWCO Amicon Ultra-15 Centrifugal Filter (Millipore Sigma) unit to remove yeast extract stock solution components larger than 3,000 Da. The resulting solutions were filter-sterilized and used to supplement minimal media at the indicated concentrations.

### Fluorescent time-lapse microscopy

Strains of interest were grown overnight in 5 mL of LB liquid broth shaking at 37°C to obtain a saturated culture. Cultures were back diluted 1,000-fold and grown to an early exponential phase (approximately 3 hours). Cells were concentrated by centrifugation, when necessary, at 5,000 *g* for 1 minute, before being resuspended in 100 µL of media. Cells were immobilized on agarose pads by spotting 0.5 µL of concentrated culture on the pad, then inverting the pad onto a glass bottom petri dish for imaging. Agarose pads for microscopy were constructed out of LB or MOPS minimal medium containing 1.5% agarose (Thermo Fisher cat. #16500500). Imaging was performed using equipment available at the University of Alabama at Birmingham High-Resolution Imaging Facility (HRIF); a Nikon Ti2 inverted fluorescence microscope with a tandem galvano and Nikon A1R-HD25 resonance scanner up to 30 1,024 × 1,024 images/s.

Tokai Hit incubation stage chamber was used to heat samples and objectives to 37°C to facilitate the growth of bacteria for live imaging. Images were captured at 60× or 100× magnification in both transmitted light differential interference contrast image and GFP or appropriate fluorescent channels as needed. Automated time-lapse imaging was performed at 37°C, and motorized x, y, and z tracking was controlled and automated by acquisition software Nis Elements 5.0 Imaging Software available in the HRIF at UAB. Analysis of microscopy images captured will be analyzed in FIJI (**F**iji **I**s **J**ust **I**mageJ) for cell length, fluorescence quantification, and tracking (145–147).

1 mM Isopropyl β-d-1-thiogalactopyranoside (IPTG) was added to cells containing the λ*attB::P_lac_-gfp-ftsZ* reporter. 1 mM IPTG was also added to the agarose pad for imaging during the time-lapse of these strains.

### Cell length quantification and FtsZ-Ring positioning

Length of cells was determined and manually calculated using FIJI’s line tool and measuring length with built-in measurements tool in FIJI after setting the appropriate scale. FtsZ-ring position was determined by manually selecting and designating the pole of each cell quantified with the line segment tool in FIJI, and measuring the distance between the two poles of the cell after the scale was set. Poles were arbitrarily designated as either 0 or 1. The FtsZ-ring was manually determined, and the distance from the pole was measured and then divided by the total length of the cell (119). The fraction of cells that developed into branching cells or had multiple FtsZ-rings forming within a single cell as well as disrupted FtsZ-ring placement (not central to the cell) was determined from direct visual observation and cell counting from time-lapse microscopy. Cells were grown on LB agarose pads at 37°C for 3 hours, and when branched cells formed, time-lapse was stopped and both the total number of cells and the number of cells that were branched were counted.

### Cryo-electron microscopy

Cryo-electron microscopy was performed with the help of the UAB institutional Research Core Program with the help of Dr. Terje Dokland and Dr. James Kizziah (University of Alabama at Birmingham). A Thermo Fisher Scientific (TFS) Glacios 2 equipped with a Falcon 4i direct electron detector is optimized for high-throughput cryo-EM and ease of use *via* the TFS EPU and Tomography software. It has demonstrated data collection speeds up to ~500 images/hour and a capability of 2.2 Å resolution *via* single particle analysis with apoferritin. A TFS Talos F200C equipped with a Ceta-S CMOS camera and a Direct Electron Apollo direct electron detector is used for imaging of negatively stained samples and specialized cryo-EM applications.

For sample preparation, a Pelco EasiGlow glow discharge machine, a PIE Scientific TergeoEM plasma cleaner, and FEI Vitrobot Mark IV sample vitrification robot were used. Gatan 626 and 698 “Elsa” cryo-holders are used for cryo-EM on the Talos F200C. An in-house GPU-accelerated computing workstation is used for on-the-fly processing of single particle cryo-EM data with CryoSPARC Live, and the CEMF has a direct 10Gbps fiber link to the UAB supercomputer for offloading and distribution of data to users. Cells were grown overnight until the morning when they were back diluted and grown for 3 hours until an OD_600_ of 0.1 was reached, and cells were centrifuged at 8,000 *g* for 2 minutes and resuspended in PBS for CEM sample prep.

### Transmission electron microscopy

TEM was performed at/by the High-Resolution Imaging Facility (HRIF) at the University of Alabama at Birmingham. Wild-type MG1655 (MJG0001) and *ppk relA spoT* (MJG1282) were grown in LB at 37°C until exponential phase growth (approximately OD_600_ 0.3–0.5). Cells were then spun down and collected. Remove media from pellet and fix in 1% Osmium tetroxide (148) in 0.1M Sodium Cacodylate Buffer pH 7.4 at room temperature in the dark for 1 hour, then 3 times 0.1M Sodium Cacodylate Buffer pH 7.4 rinse for 15 minutes each. 1% Low molecular weight tannic acid (Ted Pella Inc) for 20 minutes, 3 times 0.1M Sodium Cacodylate Buffer pH 7.4 rinse for 15 minutes each.

The specimens were dehydrated through a series of graded ethyl alcohols from 50% to 100%. The schedule was as follows: 50% for 5 minutes, 2% uranyl acetate in 50% EtOH for 30 minutes in the dark, 50% for 5 minutes, 80% for 5 minutes, 95% for 5 min, and four changes of 100% for 15 minutes each. After dehydration, the infiltration process requires steps through an intermediate solvent, 2 changes of 100% propylene oxide (P.O.) for 10 minutes each and finally into a 50:50 mixture of P.O. and the embedding resin (Embed 812, Electron Microscopy Sciences, Hatfield, PA) for 12–18 hours.

The specimen was transferred to fresh 100% embedding media. The following day the specimen was then embedded in a fresh change of 100% embedding media. Blocks were polymerized overnight in a 60 degree C embedding oven and are then ready to section.

### Procedure to a section for transmission electron microscopy

The resin blocks are first thick sectioned at 0.5–1 microns with a diamond histo knife using an ultramicrotome and sections are stained with Toluidine Blue, these sections are used as a reference to trim blocks for thin sectioning. The appropriate blocks are then thin sectioned using a diamond knife (Diatome, Electron Microscopy Sciences, Fort Washington, PA)) at 70–100 nm (silver to pale gold using color interference) and sections are then placed on either copper or nickel mesh grids. After drying, the sections are stained with heavy metals, uranyl acetate, and lead citrate for contrast. After drying the grids are then viewed on a JEOL 1400 FLASH 120kv TEM (JEOL USA Inc., Peabody, MA). Digital images are taken with an AMT NanoSprint43 Mark II camera (AMT Imaging, Woburn, MA) and transferred *via* UAB BOX or other devices.

### Statistical analyses

We used GraphPad Prism version 10.2.2 for Macintosh (GraphPad Software) to perform all statistical analyses and graph generation.

## Data Availability

All strains generated in the course of this work are available from the authors upon request. We deposited DNA sequencing data in the NIH Sequence Read Archive (accession number: PRJNA1032912), and all other raw data are available on FigShare (DOI: 10.6084/m9.figshare.c.7430740).

## SUPPLEMENTAL MATERIAL

Supplemental material mbio.03511-24-s0001.pdf Figures S1 to S16, Table S1, and legends for supplemental videos.

Video SV1 mbio.03511-24-s0002.avi Time-lapse fluorescent microscopy of wild-type MG1655 (MJG0001) on MOPS minimal media agarose pad at 37°C.

Video SV2 mbio.03511-24-s0003.avi Time-lapse fluorescent microscopy of *ppk relA* mutant (MJG2403) with FtsZ-GFP reporter on MOPS minimal media agarose pad at 37°C.

Video SV3 mbio.03511-24-s0004.avi Time-lapse fluorescent microscopy of *ppk relA* spoT mutant (MJG2405) with FtsZ-GFP reporter on MOPS minimal media agarose pad at 37°C.

Video SV4 mbio.03511-24-s0005.avi Time-lapse fluorescent microscopy of wild-type MG1655 (MJG2401) with FtsZ-GFP reporter on LB media agarose pad at 37°C.

Video SV5 mbio.03511-24-s0006.avi Time-lapse fluorescent microscopy of *ppk relA* FtsZ-GFP (MJG2403) mutant on LB agarose pad at 37°C.This video has six Z-rings within a single growing cell. Fig 5B is a still image from this video.

Video SV6 mbio.03511-24-s0007.avi Time-lapse fluorescent microscopy of *ppk relA* FtsZ-GFP (MJG2403) mutant on LB agarose pad at 37°C showing branching cells developing, as shown in still images in Fig 7.

Video SV7 mbio.03511-24-s0008.avi Time-lapse fluorescent microscopy of *ppk relA* spoT FtsZ-GFP (MJG2405) mutant on LB agarose pad at 37°C showing branching cells developing.

Video SV8 mbio.03511-24-s0009.avi Time-lapse fluorescent microscopy of *ppk relA* spoT FtsZ-GFP (MJG2405) mutant on LB agarose pad at 37°C showing branching cells and a spheroplast developing in the lower left corner of the video.

Video SV9 mbio.03511-24-s0010.avi Time-lapse microscopy of ∆*ppk* ∆*relA* spoT *rpoB3443* (MJG1581) on LB agarose pad at 37°C, imaged every 10 minutes.

ASM does not own the copyrights to Supplemental Material that may be linked to, or accessed through, an article. The authors have granted ASM a non-exclusive, world-wide license to publish the Supplemental Material files. Please contact the corresponding author directly for reuse.

## References

[1] Dalebroux ZD, Swanson MS. 2012. ppGpp: magic beyond RNA polymerase. Nat Rev Microbiol 10:203–212. doi:10.1038/nrmicro2720 22337166 PMC13198741

[2] Potrykus K, Cashel M. 2008. (p)ppGpp: still magical? Annu Rev Microbiol 62:35–51. doi:10.1146/annurev.micro.62.081307.162903 18454629

[3] Rao NN, Gómez-García MR, Kornberg A. 2009. Inorganic polyphosphate: essential for growth and survival. Annu Rev Biochem 78:605–647. doi:10.1146/annurev.biochem.77.083007.093039 19344251

[4] Bowlin MQ, Gray MJ. 2021. Inorganic polyphosphate in host and microbe biology. Trends Microbiol 29:1013–1023. doi:10.1016/j.tim.2021.02.002 33632603 PMC8380261

[5] Jain V, Kumar M, Chatterji D. 2006. ppGpp: stringent response and survival. J Microbiol 44:1–10. 16554711

[6] Magnusson LU, Farewell A, Nyström T. 2005. ppGpp: a global regulator in Escherichia coli. Trends Microbiol 13:236–242. doi:10.1016/j.tim.2005.03.008 15866041

[7] Faxén M, Isaksson LA. 1994. Functional interactions between translation, transcription and ppGpp in growing Escherichia coli. Biochim et Biophys Acta (BBA) - Gene Struct and Express 1219:425–434. doi:10.1016/0167-4781(94)90068-X 7918639

[8] Atkinson GC, Tenson T, Hauryliuk V. 2011. The RelA/SpoT homolog (RSH) superfamily: distribution and functional evolution of ppGpp synthetases and hydrolases across the tree of life. PLoS ONE 6:e23479. doi:10.1371/journal.pone.0023479 21858139 PMC3153485

[9] Irving SE, Corrigan RM. 2018. Triggering the stringent response: signals responsible for activating (p)ppGpp synthesis in bacteria. Microbiol (Reading, Engl) 164:268–276. doi:10.1099/mic.0.000621 29493495

[10] Arenz S, Abdelshahid M, Sohmen D, Payoe R, Starosta AL, Berninghausen O, Hauryliuk V, Beckmann R, Wilson DN. 2016. The stringent factor RelA adopts an open conformation on the ribosome to stimulate ppGpp synthesis. Nucleic Acids Res 44:6471–6481. doi:10.1093/nar/gkw470 27226493 PMC5291266

[11] Loveland AB, Bah E, Madireddy R, Zhang Y, Brilot AF, Grigorieff N, Korostelev AA. 2016. Ribosome*RelA structures reveal the mechanism of stringent response activation. Elife 5:e17029. doi:10.7554/eLife.17029 27434674 PMC4974054

[12] Brown A, Fernández IS, Gordiyenko Y, Ramakrishnan V. 2016. Ribosome-dependent activation of stringent control. Nature New Biol 534:277–280. doi:10.1038/nature17675 PMC4900451 27279228

[13] Germain E, Guiraud P, Byrne D, Douzi B, Djendli M, Maisonneuve E. 2019. YtfK activates the stringent response by triggering the alarmone synthetase SpoT in Escherichia coli. Nat Commun 10:5763. doi:10.1038/s41467-019-13764-4 31848343 PMC6917717

[14] Davis RT, Brown PD. 2020. spoT-mediated stringent response influences environmental and nutritional stress tolerance, biofilm formation and antimicrobial resistance in Klebsiella pneumoniae. APMIS 128:48–60. doi:10.1111/apm.13006 31693234

[15] Hua Xiao MK, Ikehara K, Zemel S, Glaser G, Cashel M. 1990. Residual guanosine 3’,5’-bispyrophosphate synthetic activity of relA null mutants can be eliminated by spoT null mutations. J Biol Chem 266:5980–5990. 2005134

[16] Kari C, Török I, Travers A. 1977. ppGpp cycle in Escherichia coli. Mol Gen Genet 150:249–255. doi:10.1007/BF00268123 321933

[17] Gallant J, Margason G, Finch B. 1972. On the turnover of ppGpp in Escherichia coli. J Biol Chem 247:6055–6058. doi:10.1016/S0021-9258(19)44762-5 4568601

[18] Sanyal R, Harinarayanan R. 2020. Activation of RelA by pppGpp as the basis for its differential toxicity over ppGpp in Escherichia coli. J Biosci 45:28. 32020910

[19] Kushwaha GS, Oyeyemi BF, Bhavesh NS. 2019. Stringent response protein as a potential target to intervene persistent bacterial infection. Biochimie 165:67–75. doi:10.1016/j.biochi.2019.07.006 31302165

[20] An G, Justesen J, Watson RJ, Friesen JD. 1979. Cloning the spoT gene of Escherichia coli: identification of the spoT gene product. J Bacteriol 137:1100–1110. doi:10.1128/jb.137.3.1100-1110.1979 374338 PMC218288

[21] Sarubbi E, Rudd KE, Cashel M. 1988. Basal ppGpp level adjustment shown by new spoT mutants affect steady state growth rates and rrnA ribosomal promoter regulation in Escherichia coli. Mol Gen Genet 213:214–222. doi:10.1007/BF00339584 2460731

[22] Cozzone AJ. 1980. Stringent control and protein synthesis in bacteria. Biochimie 62:647–664. doi:10.1016/s0300-9084(80)80022-8 7004494

[23] Zuo Y, Wang Y, Steitz TA. 2013. The mechanism of E. coli RNA polymerase regulation by ppGpp is suggested by the structure of their complex. Mol Cell 50:430–436. doi:10.1016/j.molcel.2013.03.020 23623685 PMC3677725

[24] Ross W, Sanchez-Vazquez P, Chen AY, Lee JH, Burgos HL, Gourse RL. 2016. ppGpp binding to a site at the RNAP-DksA interface accounts for its dramatic effects on transcription initiation during the stringent response. Mol Cell 62:811–823. doi:10.1016/j.molcel.2016.04.029 27237053 PMC4912440

[25] Zhai Y, Minnick PJ, Pribis JP, Garcia-Villada L, Hastings PJ, Herman C, Rosenberg SM. 2023. ppGpp and RNA-polymerase backtracking guide antibiotic-induced mutable gambler cells. Mol Cell 83:1298–1310. doi:10.1016/j.molcel.2023.03.003 36965481 PMC10317147

[26] Wang B, Grant RA, Laub MT. 2020. ppGpp coordinates nucleotide and amino-acid synthesis in E. coli during starvation. Mol Cell 80:29–42. doi:10.1016/j.molcel.2020.08.005 32857952 PMC8362273

[27] Hobbs JK, Boraston AB. 2019. (p)ppGpp and the stringent response: an emerging threat to antibiotic therapy. ACS Infect Dis 5:1505–1517. doi:10.1021/acsinfecdis.9b00204 31287287

[28] Mwangi MM, Kim C, Chung M, Tsai J, Vijayadamodar G, Benitez M, Jarvie TP, Du L, Tomasz A. 2013. Whole-genome sequencing reveals a link between β-lactam resistance and synthetases of the alarmone (p)ppGpp in Staphylococcus aureus. Microb Drug Resist 19:153–159. doi:10.1089/mdr.2013.0053 23659600 PMC3662374

[29] Strugeon E, Tilloy V, Ploy M-C, Da Re S. 2016. The stringent response promotes antibiotic resistance dissemination by regulating integron integrase expression in biofilms. MBio 7:e00868-16. doi:10.1128/mBio.00868-16 27531906 PMC4992968

[30] Aedo S, Tomasz A. 2016. Role of the stringent stress response in the antibiotic resistance phenotype of methicillin-resistant Staphylococcus aureus. Antimicrob Agents Chemother 60:2311–2317. doi:10.1128/AAC.02697-15 26833147 PMC4808156

[31] Steinchen W, Bange G. 2016. The magic dance of the alarmones (p)ppGpp. Mol Microbiol 101:531–544. doi:10.1111/mmi.13412 27149325

[32] Dalebroux ZD, Svensson SL, Gaynor EC, Swanson MS. 2010. ppGpp conjures bacterial virulence. Microbiol Mol Biol Rev 74:171–199. doi:10.1128/MMBR.00046-09 20508246 PMC2884408

[33] Gaca AO, Colomer-Winter C, Lemos JA. 2015. Many means to a common end: the intricacies of (p)ppGpp metabolism and its control of bacterial homeostasis. J Bacteriol 197:1146–1156. doi:10.1128/JB.02577-14 25605304 PMC4352657

[34] Boutte CC, Crosson S. 2013. Bacterial lifestyle shapes stringent response activation. Trends Microbiol 21:174–180. doi:10.1016/j.tim.2013.01.002 23419217 PMC4238387

[35] Cashel M, Gallant J. 1969. Two compounds implicated in the function of the RC gene of Escherichia coli. Nature New Biol 221:838–841. doi:10.1038/221838a0 4885263

[36] Büke F, Grilli J, Cosentino Lagomarsino M, Bokinsky G, Tans SJ. 2022. ppGpp is a bacterial cell size regulator. Curr Biol 32:870–877. doi:10.1016/j.cub.2021.12.033 34990598

[37] Schreiber G, Metzger S, Aizenman E, Roza S, Cashel M, Glaser G. 1991. Overexpression of the relA gene in Escherichia coli. J Biol Chem 266:3760–3767. 1899866

[38] Ferullo DJ, Lovett ST. 2008. The stringent response and cell cycle arrest in Escherichia coli. PLoS Genet 4:e1000300. doi:10.1371/journal.pgen.1000300 19079575 PMC2586660

[39] Singh V, Harinarayanan R. 2024. (p)ppGpp buffers cell division when membrane fluidity decreases in Escherichia coli. Mol Microbiol. doi:10.1111/mmi.15323 39461000

[40] Kulaev IS, Vagabov VM. 1983. Polyphosphate metabolism in micro-organisms. Adv Microb Physiol 24:83–171. doi:10.1016/s0065-2911(08)60385-9 6320606

[41] Gray MJ, Wholey W-Y, Wagner NO, Cremers CM, Mueller-Schickert A, Hock NT, Krieger AG, Smith EM, Bender RA, Bardwell JCA, Jakob U. 2014. Polyphosphate is a primordial chaperone. Mol Cell 53:689–699. doi:10.1016/j.molcel.2014.01.012 24560923 PMC3996911

[42] Gray MJ. 2020. Interactions between DksA and stress-responsive alternative sigma factors control inorganic polyphosphate accumulation in Escherichia coli. J Bacteriol 202:e00133-20. doi:10.1128/JB.00133-20 32341074 PMC7317045

[43] Gray MJ. 2019. Inorganic polyphosphate accumulation in Escherichia coli is regulated by DksA but not by (p)ppGpp. J Bacteriol 201:e00664-18. doi:10.1128/JB.00664-18 30745375 PMC6456864

[44] Rao NN, Liu S, Kornberg A. 1998. Inorganic polyphosphate in Escherichia coli: the phosphate regulon and the stringent response. J Bacteriol 180:2186–2193. doi:10.1128/JB.180.8.2186-2193.1998 9555903 PMC107147

[45] Kornberg A, Rao NN, Ault-Riché D. 1999. Inorganic polyphosphate: a molecule of many functions. Annu Rev Biochem 68:89–125. doi:10.1146/annurev.biochem.68.1.89 10872445

[46] Brown MRW, Kornberg A. 2004. Inorganic polyphosphate in the origin and survival of species. Proc Natl Acad Sci U S A 101:16085–16087. doi:10.1073/pnas.0406909101 15520374 PMC528972

[47] Ault-Riché D, Fraley CD, Tzeng CM, Kornberg A. 1998. Novel assay reveals multiple pathways regulating stress-induced accumulations of inorganic polyphosphate in Escherichia coli. J Bacteriol 180:1841–1847. doi:10.1128/JB.180.7.1841-1847.1998 9537383 PMC107098

[48] Kim KS, Rao NN, Fraley CD, Kornberg A. 2002. Inorganic polyphosphate is essential for long-term survival and virulence factors in Shigella and Salmonella spp. Proc Natl Acad Sci U S A 99:7675–7680. doi:10.1073/pnas.112210499 12032342 PMC124319

[49] Ogawa N, Tzeng CM, Fraley CD, Kornberg A. 2000. Inorganic polyphosphate in Vibrio cholerae: genetic, biochemical, and physiologic features. J Bacteriol 182:6687–6693. doi:10.1128/JB.182.23.6687-6693.2000 11073913 PMC111411

[50] Ortiz-Severín J, Varas M, Bravo-Toncio C, Guiliani N, Chávez FP. 2015. Multiple antibiotic susceptibility of polyphosphate kinase mutants (ppk1 and ppk2) from Pseudomonas aeruginosa PAO1 as revealed by global phenotypic analysis. Biol Res 48:22. doi:10.1186/s40659-015-0012-0 25907584 PMC4424552

[51] Rashid MH, Rumbaugh K, Passador L, Davies DG, Hamood AN, Iglewski BH, Kornberg A. 2000. Polyphosphate kinase is essential for biofilm development, quorum sensing, and virulence of Pseudomonas aeruginosa. Proc Natl Acad Sci U S A 97:9636–9641. doi:10.1073/pnas.170283397 10931957 PMC16917

[52] Peng L, Luo WY, Zhao T, Wan CS, Jiang Y, Chi F, Zhao W, Cao H, Huang SH. 2012. Polyphosphate kinase 1 is required for the pathogenesis process of meningitic Escherichia coli K1 (RS218). Future Microbiol 7:411–423. doi:10.2217/fmb.12.3 22393893

[53] Kornberg A. 1995. Inorganic polyphosphate: toward making a forgotten polymer unforgettable. J Bacteriol 177:491–496. doi:10.1128/jb.177.3.491-496.1995 7836277 PMC176618

[54] Akiyama M, Crooke E, Kornberg A. 1992. The polyphosphate kinase gene of Escherichia coli. Isolation and sequence of the ppk gene and membrane location of the protein. J Biol Chem 267:22556–22561. doi:10.1016/S0021-9258(18)41708-5 1331061

[55] Akiyama M, Crooke E, Kornberg A. 1993. An exopolyphosphatase of Escherichia coli. The enzyme and its ppx gene in a polyphosphate operon. J Biol Chem 268:633–639. doi:10.1016/S0021-9258(18)54198-3 8380170

[56] Chugh S, Tiwari P, Suri C, Gupta SK, Singh P, Bouzeyen R, Kidwai S, Srivastava M, Rameshwaram NR, Kumar Y, Asthana S, Singh R. 2024. Polyphosphate kinase-1 regulates bacterial and host metabolic pathways involved in pathogenesis of Mycobacterium tuberculosis. Proc Natl Acad Sci U S A 121:e2309664121. doi:10.1073/pnas.2309664121 38170746 PMC10786269

[57] Chuang YM, Belchis DA, Karakousis PC. 2013. The polyphosphate kinase gene ppk2 is required for Mycobacterium tuberculosis inorganic polyphosphate regulation and virulence. MBio 4:e00039-13. doi:10.1128/mBio.00039-13 23695835 PMC3663568

[58] Neville N, Roberge N, Jia Z. 2022. Polyphosphate kinase 2 (PPK2) enzymes: structure, function, and roles in bacterial physiology and virulence. Int J Mol Sci 23:670. doi:10.3390/ijms23020670 35054854 PMC8776046

[59] Parks QM, Hobden JA. 2005. Polyphosphate kinase 1 and the ocular virulence of Pseudomonas aeruginosa. Invest Ophthalmol Vis Sci 46:248–251. doi:10.1167/iovs.04-0340 15623780

[60] Kuroda A, Tanaka S, Ikeda T, Kato J, Takiguchi N, Ohtake H. 1999. Inorganic polyphosphate kinase is required to stimulate protein degradation and for adaptation to amino acid starvation in Escherichia coli. Proc Natl Acad Sci U S A 96:14264–14269. doi:10.1073/pnas.96.25.14264 10588694 PMC24425

[61] Bai K, Yan H, Chen X, Lyu Q, Jiang N, Li J, Luo L. 2021. The role of RelA and SpoT on ppGpp production, stress response, growth regulation, and pathogenicity in Xanthomonas campestris pv. campestris. Microbiol Spectr 9:e0205721. doi:10.1128/spectrum.02057-21 34935430 PMC8693919

[62] Kuroda A, Murphy H, Cashel M, Kornberg A. 1997. Guanosine tetra- and pentaphosphate promote accumulation of inorganic polyphosphate in Escherichia coli. J Biol Chem 272:21240–21243. doi:10.1074/jbc.272.34.21240 9261133

[63] Gourse RL, Chen AY, Gopalkrishnan S, Sanchez-Vazquez P, Myers A, Ross W. 2018. Transcriptional responses to ppGpp and DksA. Annu Rev Microbiol 72:163–184. doi:10.1146/annurev-micro-090817-062444 30200857 PMC6586590

[64] Cohen H, Adani B, Cohen E, Piscon B, Azriel S, Desai P, Bähre H, McClelland M, Rahav G, Gal-Mor O. 2022. The ancestral stringent response potentiator, DksA has been adapted throughout Salmonella evolution to orchestrate the expression of metabolic, motility, and virulence pathways. Gut Microbes 14:1997294. doi:10.1080/19490976.2021.1997294 34923900 PMC8726615

[65] Paul BJ, Barker MM, Ross W, Schneider DA, Webb C, Foster JW, Gourse RL. 2004. DksA: a critical component of the transcription initiation machinery that potentiates the regulation of rRNA promoters by ppGpp and the initiating NTP. Cell 118:311–322. doi:10.1016/j.cell.2004.07.009 15294157

[66] Wang B, Dai P, Ding D, Del Rosario A, Grant RA, Pentelute BL, Laub MT. 2019. Affinity-based capture and identification of protein effectors of the growth regulator ppGpp. Nat Chem Biol 15:141–150. doi:10.1038/s41589-018-0183-4 30559427 PMC6366861

[67] Potrykus K, Murphy H, Philippe N, Cashel M. 2011. ppGpp is the major source of growth rate control in E. coli. Environ Microbiol 13:563–575. doi:10.1111/j.1462-2920.2010.02357.x 20946586 PMC4556285

[68] Rutherford ST, Villers CL, Lee JH, Ross W, Gourse RL. 2009. Allosteric control of Escherichia coli rRNA promoter complexes by DksA. Genes Dev 23:236–248. doi:10.1101/gad.1745409 19171784 PMC2648540

[69] Murphy H, Cashel M. 2003. Isolation of RNA polymerase suppressors of a (p)ppGpp deficiency. Methods Enzymol 371:596–601. doi:10.1016/S0076-6879(03)71044-1 14712731

[70] Zhou YN, Jin DJ. 1998. The rpoB mutants destabilizing initiation complexes at stringently controlled promoters behave like "stringent" RNA polymerases in Escherichia coli. Proc Natl Acad Sci U S A 95:2908–2913. doi:10.1073/pnas.95.6.2908 9501189 PMC19668

[71] Bartlett MS, Gaal T, Ross W, Gourse RL. 1998. RNA polymerase mutants that destabilize RNA polymerase-promoter complexes alter NTP-sensing by rrn P1 promoters. J Mol Biol 279:331–345. doi:10.1006/jmbi.1998.1779 9642041

[72] Hernandez VJ, Cashel M. 1995. Changes in conserved region 3 of Escherichia coli sigma 70 mediate ppGpp-dependent functions in vivo. J Mol Biol 252:536–549. doi:10.1006/jmbi.1995.0518 7563072

[73] Angelini S, My L, Bouveret E. 2012. Disrupting the Acyl Carrier Protein/SpoT interaction in vivo: identification of ACP residues involved in the interaction and consequence on growth. PLoS ONE 7:e36111. doi:10.1371/journal.pone.0036111 22558350 PMC3340395

[74] Xiao H, Kalman M, Ikehara K, Zemel S, Glaser G, Cashel M. 1991. Residual guanosine 3',5'-bispyrophosphate synthetic activity of relA null mutants can be eliminated by spoT null mutations. J Biol Chem 266:5980–5990. 2005134

[75] Irving SE, Choudhury NR, Corrigan RM. 2021. The stringent response and physiological roles of (pp)pGpp in bacteria. Nat Rev Microbiol 19:256–271. doi:10.1038/s41579-020-00470-y 33149273

[76] Bange G, Brodersen DE, Liuzzi A, Steinchen W. 2021. Two P or Not Two P: understanding regulation by the bacterial second messengers (P)ppGpp. Annu Rev Microbiol 75:383–406. doi:10.1146/annurev-micro-042621-122343 34343020

[77] Crooke E, Akiyama M, Rao NN, Kornberg A. 1994. Genetically altered levels of inorganic polyphosphate in Escherichia coli. J Biol Chem 269:6290–6295. 8119977

[78] Elbing KL, Brent R. 2019. Recipes and tools for culture of Escherichia coli. Curr Protoc Mol Biol 125:e83. doi:10.1002/cpmb.83 30412361 PMC6819147

[79] Neidhardt FC, Bloch PL, Smith DF. 1974. Culture medium for enterobacteria. J Bacteriol 119:736–747. doi:10.1128/jb.119.3.736-747.1974 4604283 PMC245675

[80] Lange R, Fischer D, Hengge-Aronis R. 1995. Identification of transcriptional start sites and the role of ppGpp in the expression of rpoS, the structural gene for the sigma S subunit of RNA polymerase in Escherichia coli. J Bacteriol 177:4676–4680. doi:10.1128/jb.177.16.4676-4680.1995 7642494 PMC177232

[81] Bouillet S, Bauer TS, Gottesman S. 2024. RpoS and the bacterial general stress response. Microbiol Mol Biol Rev 88:e0015122. doi:10.1128/mmbr.00151-22 38411096 PMC10966952

[82] Shiba T, Tsutsumi K, Yano H, Ihara Y, Kameda A, Tanaka K, Takahashi H, Munekata M, Rao NN, Kornberg A. 1997. Inorganic polyphosphate and the induction of rpoS expression. Proc Natl Acad Sci U S A 94:11210–11215. doi:10.1073/pnas.94.21.11210 9326588 PMC23418

[83] Boehm A, Steiner S, Zaehringer F, Casanova A, Hamburger F, Ritz D, Keck W, Ackermann M, Schirmer T, Jenal U. 2009. Second messenger signalling governs Escherichia coli biofilm induction upon ribosomal stress. Mol Microbiol 72:1500–1516. doi:10.1111/j.1365-2958.2009.06739.x 19460094

[84] Bowlin MQ, Lieber AD, Long AR, Gray MJ. 2024. C-terminal poly-histidine tags alter Escherichia coli polyphosphate kinase activity and susceptibility to inhibition. J Mol Biol 436:168651. doi:10.1016/j.jmb.2024.168651 38866092 PMC11297678

[85] Rudat AK, Pokhrel A, Green TJ, Gray MJ. 2018. Mutations in Escherichia coli polyphosphate kinase that lead to dramatically increased in vivo polyphosphate levels. J Bacteriol 200:e00697-17. doi:10.1128/JB.00697-17 29311274 PMC5826030

[86] Roghanian M, Semsey S, Løbner-Olesen A, Jalalvand F. 2019. (P)ppGpp-mediated stress response induced by defects in outer membrane biogenesis and ATP production promotes survival in Escherichia coli. Sci Rep 9:2934. doi:10.1038/s41598-019-39371-3 30814571 PMC6393671

[87] Varela C, Mauriaca C, Paradela A, Albar JP, Jerez CA, Chávez FP. 2010. New structural and functional defects in polyphosphate deficient bacteria: a cellular and proteomic study. BMC Microbiol 10:7. doi:10.1186/1471-2180-10-7 20067623 PMC2817675

[88] Mueller EA, Westfall CS, Levin PA. 2020. pH-dependent activation of cytokinesis modulates Escherichia coli cell size. PLoS Genet 16:e1008685. doi:10.1371/journal.pgen.1008685 32203516 PMC7117782

[89] Bi E, Dai K, Subbarao S, Beall B, Lutkenhaus J. 1991. FtsZ and cell division. Res Microbiol 142:249–252. doi:10.1016/0923-2508(91)90037-b 1925024

[90] Silber N, Matos de Opitz CL, Mayer C, Sass P. 2020. Cell division protein FtsZ: from structure and mechanism to antibiotic target. Future Microbiol 15:801–831. doi:10.2217/fmb-2019-0348 32692252

[91] Mahone CR, Goley ED. 2020. Bacterial cell division at a glance. J Cell Sci 133. doi:10.1242/jcs.237057 PMC7157936 32269092

[92] de Pedro MA, Young KD, Höltje J-V, Schwarz H. 2003. Branching of Escherichia coli cells arises from multiple sites of inert peptidoglycan. J Bacteriol 185:1147–1152. doi:10.1128/JB.185.4.1147-1152.2003 12562782 PMC142844

[93] Shi H, Bratton BP, Gitai Z, Huang KC. 2018. How to build a bacterial cell: MreB as the foreman of E. coli construction. Cell 172:1294–1305. doi:10.1016/j.cell.2018.02.050 29522748 PMC5846203

[94] Rojas ER, Huang KC. 2018. Regulation of microbial growth by turgor pressure. Curr Opin Microbiol 42:62–70. doi:10.1016/j.mib.2017.10.015 29125939

[95] Scheie PO, Rehberg R. 1972. Response of Escherichia coli B-r to high concentrations of sucrose in a nutrient medium. J Bacteriol 109:229–235. doi:10.1128/jb.109.1.229-235.1972 4550664 PMC247271

[96] Wong F, Stokes JM, Cervantes B, Penkov S, Friedrichs J, Renner LD, Collins JJ. 2021. Cytoplasmic condensation induced by membrane damage is associated with antibiotic lethality. Nat Commun 12:2321. doi:10.1038/s41467-021-22485-6 33875652 PMC8055701

[97] Jin DJ, Gross CA. 1989. Characterization of the pleiotropic phenotypes of rifampin-resistant rpoB mutants of Escherichia coli. J Bacteriol 171:5229–5231. doi:10.1128/jb.171.9.5229-5231.1989 2670912 PMC210350

[98] Walukiewicz HE, Farris Y, Burnet MC, Feid SC, You Y, Kim H, Bank T, Christensen D, Payne SH, Wolfe AJ, Rao CV, Nakayasu ES. 2024. Regulation of bacterial stringent response by an evolutionarily conserved ribosomal protein L11 methylation. MBio. doi:10.1128/mbio.01773-24:e0177324 PMC11481523 39189746

[99] Sanchez-Vazquez P, Dewey CN, Kitten N, Ross W, Gourse RL. 2019. Genome-wide effects on Escherichia coli transcription from ppGpp binding to its two sites on RNA polymerase. Proc Natl Acad Sci U S A 116:8310–8319. doi:10.1073/pnas.1819682116 30971496 PMC6486775

[100] Durfee T, Hansen AM, Zhi H, Blattner FR, Jin DJ. 2008. Transcription profiling of the stringent response in Escherichia coli. J Bacteriol 190:1084–1096. doi:10.1128/JB.01092-07 18039766 PMC2223561

[101] Rajagopala SV, Sikorski P, Kumar A, Mosca R, Vlasblom J, Arnold R, Franca-Koh J, Pakala SB, Phanse S, Ceol A, Häuser R, Siszler G, Wuchty S, Emili A, Babu M, Aloy P, Pieper R, Uetz P. 2014. The binary protein-protein interaction landscape of Escherichia coli. Nat Biotechnol 32:285–290. doi:10.1038/nbt.2831 24561554 PMC4123855

[102] Arifuzzaman M, Maeda M, Itoh A, Nishikata K, Takita C, Saito R, Ara T, Nakahigashi K, Huang HC, Hirai A, Tsuzuki K, Nakamura S, Altaf-Ul-Amin M, Oshima T, Baba T, Yamamoto N, Kawamura T, Ioka-Nakamichi T, Kitagawa M, Tomita M, Kanaya S, Wada C, Mori H. 2006. Large-scale identification of protein-protein interaction of Escherichia coli K-12. Genome Res 16:686–691. doi:10.1101/gr.4527806 16606699 PMC1457052

[103] Butland G, Peregrín-Alvarez JM, Li J, Yang W, Yang X, Canadien V, Starostine A, Richards D, Beattie B, Krogan N, Davey M, Parkinson J, Greenblatt J, Emili A. 2005. Interaction network containing conserved and essential protein complexes in Escherichia coli. Nature New Biol 433:531–537. doi:10.1038/nature03239 15690043

[104] Blum E, Py B, Carpousis AJ, Higgins CF. 1997. Polyphosphate kinase is a component of the Escherichia coli RNA degradosome. Mol Microbiol 26:387–398. doi:10.1046/j.1365-2958.1997.5901947.x 9383162

[105] Gross MH, Konieczny I. 2020. Polyphosphate induces the proteolysis of ADP-bound fraction of initiator to inhibit DNA replication initiation upon stress in Escherichia coli. Nucleic Acids Res 48:5457–5466. doi:10.1093/nar/gkaa217 32282902 PMC7261185

[106] Kuroda A, Nomura K, Ohtomo R, Kato J, Ikeda T, Takiguchi N, Ohtake H, Kornberg A. 2001. Role of inorganic polyphosphate in promoting ribosomal protein degradation by the Lon protease in E. coli. Science 293:705–708. doi:10.1126/science.1061315 11474114

[107] Osbourne DO, Soo VWC, Konieczny I, Wood TK. 2014. Polyphosphate, cyclic AMP, guanosine tetraphosphate, and c-di-GMP reduce in vitro Lon activity. Bioengineered 5:264–268. doi:10.4161/bioe.29261 24874800 PMC4143397

[108] Kuroda A, Nomura K, Takiguchi N, Kato J, Ohtake H. 2006. Inorganic polyphosphate stimulates lon-mediated proteolysis of nucleoid proteins in Escherichia coli. Cell Mol Biol 52:23–29. 17543195

[109] Beaufay F, Amemiya HM, Guan J, Basalla J, Meinen BA, Chen Z, Mitra R, Bardwell JCA, Biteen JS, Vecchiarelli AG, Freddolino PL, Jakob U. 2021. Polyphosphate drives bacterial heterochromatin formation. Sci Adv 7:eabk0233. doi:10.1126/sciadv.abk0233 34936433 PMC10954037

[110] Varas M, Valdivieso C, Mauriaca C, Ortíz-Severín J, Paradela A, Poblete-Castro I, Cabrera R, Chávez FP. 2017. Multi-level evaluation of Escherichia coli polyphosphate related mutants using global transcriptomic, proteomic and phenomic analyses. Biochim Biophys Acta Gen Subj 1861:871–883. doi:10.1016/j.bbagen.2017.01.007 28069396

[111] Negreiros RS, Lander N, Huang G, Cordeiro CD, Smith SA, Morrissey JH, Docampo R. 2018. Inorganic polyphosphate interacts with nucleolar and glycosomal proteins in trypanosomatids. Mol Microbiol 110:973–994. doi:10.1111/mmi.14131 30230089 PMC6281790

[112] Krenzlin V, Roewe J, Strueve M, Martínez-Negro M, Sharma A, Reinhardt C, Morsbach S, Bosmann M. 2022. Bacterial-type long-chain polyphosphates bind human proteins in the phosphatidylinositol signaling pathway. Thromb Haemost 122:1943–1947. doi:10.1055/s-0042-1751280 35909349 PMC9798540

[113] Karp PD, Paley S, Caspi R, Kothari A, Krummenacker M, Midford PE, Moore LR, Subhraveti P, Gama-Castro S, Tierrafria VH, Lara P, Muñiz-Rascado L, Bonavides-Martinez C, Santos-Zavaleta A, Mackie A, Sun G, Ahn-Horst TA, Choi H, Covert MW, Collado-Vides J, Paulsen I. 2023. The EcoCyc Database (2023). EcoSal Plus 11:eesp00022023. doi:10.1128/ecosalplus.esp-0002-2023 37220074 PMC10729931

[114] Kuhn A, Koch HG, Dalbey RE. 2017. Targeting and insertion of membrane proteins. EcoSal Plus 7. doi:10.1128/ecosalplus.ESP-0012-2016 PMC11575690 28276312

[115] Czech L, Mais CN, Kratzat H, Sarmah P, Giammarinaro P, Freibert SA, Esser HF, Musial J, Berninghausen O, Steinchen W, Beckmann R, Koch HG, Bange G. 2022. Inhibition of SRP-dependent protein secretion by the bacterial alarmone (p)ppGpp. Nat Commun 13:1069. doi:10.1038/s41467-022-28675-0 35217658 PMC8881573

[116] Cameron TA, Margolin W. 2024. Insights into the assembly and regulation of the bacterial divisome. Nat Rev Microbiol 22:33–45. doi:10.1038/s41579-023-00942-x 37524757 PMC11102604

[117] Ramm B, Heermann T, Schwille P. 2019. The E. coli MinCDE system in the regulation of protein patterns and gradients. Cell Mol Life Sci 76:4245–4273. doi:10.1007/s00018-019-03218-x 31317204 PMC6803595

[118] Cho H, McManus HR, Dove SL, Bernhardt TG. 2011. Nucleoid occlusion factor SlmA is a DNA-activated FtsZ polymerization antagonist. Proc Natl Acad Sci USA 108:3773–3778. doi:10.1073/pnas.1018674108 21321206 PMC3048121

[119] Yu XC, Margolin W. 1999. FtsZ ring clusters in min and partition mutants: role of both the Min system and the nucleoid in regulating FtsZ ring localization. Mol Microbiol 32:315–326. doi:10.1046/j.1365-2958.1999.01351.x 10231488

[120] Ward JE, Lutkenhaus J, J. 1985. Overproduction of FtsZ induces minicell formation in E. coli. Cell 42:941–949. doi:10.1016/0092-8674(85)90290-9 2996784

[121] Pokhrel A, Lingo JC, Wolschendorf F, Gray MJ. 2019. Assaying for inorganic polyphosphate in bacteria. J Vis Exp, no. 143. doi:10.3791/58818 PMC6527317 30735204

[122] Fernández-Coll L, Cashel M. 2020. Possible roles for basal levels of (p)ppGpp: growth efficiency vs. surviving stress. Front Microbiol 11:592718. doi:10.3389/fmicb.2020.592718 33162969 PMC7581894

[123] Anderson SE, Vadia SE, McKelvy J, Levin PA. 2023. The transcription factor DksA exerts opposing effects on cell division depending on the presence of ppGpp. bioRxiv:Gpp. doi:10.1101/2023.05.15.540843 PMC10746185 37882534

[124] Christ JJ, Willbold S, Blank LM. 2020. Methods for the analysis of polyphosphate in the life sciences. Anal Chem 92:4167–4176. doi:10.1021/acs.analchem.9b05144 32039586

[125] Chen I-MA, Chu K, Palaniappan K, Ratner A, Huang J, Huntemann M, Hajek P, Ritter SJ, Webb C, Wu D, Varghese NJ, Reddy TBK, Mukherjee S, Ovchinnikova G, Nolan M, Seshadri R, Roux S, Visel A, Woyke T, Eloe-Fadrosh EA, Kyrpides NC, Ivanova NN. 2023. The IMG/M data management and analysis system v.7: content updates and new features. Nucleic Acids Res 51:D723–D732. doi:10.1093/nar/gkac976 36382399 PMC9825475

[126] Bertani G. 1951. Studies on lysogenesis. I. The mode of phage liberation by lysogenic Escherichia coli. J Bacteriol 62:293–300. doi:10.1128/jb.62.3.293-300.1951 14888646 PMC386127

[127] Blattner FR, Plunkett G 3rd, Bloch CA, Perna NT, Burland V, Riley M, Collado-Vides J, Glasner JD, Rode CK, Mayhew GF, Gregor J, Davis NW, Kirkpatrick HA, Goeden MA, Rose DJ, Mau B, Shao Y. 1997. The complete genome sequence of Escherichia coli K-12. Science 277:1453–1462. doi:10.1126/science.277.5331.1453 9278503

[128] Hernandez VJ, Bremer H. 1993. Characterization of RNA and DNA synthesis in Escherichia coli strains devoid of ppGpp. J Biol Chem 268:10851–10862. doi:10.1016/S0021-9258(18)82063-4 7684368

[129] Harms A, Fino C, Sørensen MA, Semsey S, Gerdes K. 2017. Prophages and growth dynamics confound experimental results with antibiotic-tolerant persister cells. MBio 8:e01964-17. doi:10.1128/mBio.01964-17 29233898 PMC5727415

[130] Norrander J, Kempe T, Messing J. 1983. Construction of improved M13 vectors using oligodeoxynucleotide-directed mutagenesis. Gene 26:101–106. doi:10.1016/0378-1119(83)90040-9 6323249

[131] Datsenko KA, Wanner BL. 2000. One-step inactivation of chromosomal genes in Escherichia coli K-12 using PCR products. Proc Natl Acad Sci U S A 97:6640–6645. doi:10.1073/pnas.120163297 10829079 PMC18686

[132] Silhavy TJ, Berman ML, Enquist LW. 1984. Experiments with gene fusions. Cold Spring Harbor Laboratory, Cold Spring Harbor, NY.

[133] Baba T, Ara T, Hasegawa M, Takai Y, Okumura Y, Baba M, Datsenko KA, Tomita M, Wanner BL, Mori H. 2006. Construction of Escherichia coli K-12 in-frame, single-gene knockout mutants: the Keio collection. Mol Syst Biol 2:0008. doi:10.1038/msb4100050 PMC1681482 16738554

[134] Sawitzke JA, Thomason LC, Costantino N, Bubunenko M, Datta S, Court DL. 2007. Recombineering: in vivo genetic engineering in E. coli, S. enterica, and beyond. Methods Enzymol 421:171–199. doi:10.1016/S0076-6879(06)21015-2 17352923

[135] Jin DJ, Gross CA. 1988. Mapping and sequencing of mutations in the Escherichia coli rpoB gene that lead to rifampicin resistance. J Mol Biol 202:45–58. doi:10.1016/0022-2836(88)90517-7 3050121

[136] Jin DJ, Cashel M, Friedman DI, Nakamura Y, Walter WA, Gross CA. 1988. Effects of rifampicin resistant rpoB mutations on antitermination and interaction with nusA in Escherichia coli. J Mol Biol 204:247–261. doi:10.1016/0022-2836(88)90573-6 2464690

[137] Jin DJ, Walter WA, Gross CA. 1988. Characterization of the termination phenotypes of rifampicin-resistant mutants. J Mol Biol 202:245–253. doi:10.1016/0022-2836(88)90455-x 3050123

[138] Huang Y, Zhang L. 2017. An in vitro single-primer site-directed mutagenesis method for use in biotechnology. Methods Mol Biol 1498:375–383. doi:10.1007/978-1-4939-6472-7_26 27709590

[139] Schneider DA, Gourse RL. 2004. Relationship between growth rate and ATP concentration in Escherichia coli: a bioassay for available cellular ATP. J Biol Chem 279:8262–8268. doi:10.1074/jbc.M311996200 14670952

[140] Hove-Jensen B, Rosenkrantz TJ, Zechel DL, Willemoës M. 2010. Accumulation of intermediates of the carbon-phosphorus lyase pathway for phosphonate degradation in phn mutants of Escherichia coli. J Bacteriol 192:370–374. doi:10.1128/JB.01131-09 19854894 PMC2798254

[141] Roghanian M, Van Nerom K, Takada H, Caballero-Montes J, Tamman H, Kudrin P, Talavera A, Dzhygyr I, Ekström S, Atkinson GC, Garcia-Pino A, Hauryliuk V. 2021. (p)ppGpp controls stringent factors by exploiting antagonistic allosteric coupling between catalytic domains. Mol Cell 81:3310–3322. doi:10.1016/j.molcel.2021.07.026 34416138

[142] Spira B, Ospino K. 2020. Diversity in E. coli (p)ppGpp levels and its consequences. Front Microbiol 11:1759. doi:10.3389/fmicb.2020.01759 32903406 PMC7434938

[143] Deitert A, Fees J, Mertens A, Nguyen Van D, Maares M, Haase H, Blank LM, Keil C. 2024. Rapid fluorescence assay for polyphosphate in yeast extracts using JC-D7. Yeast 41:593–604. doi:10.1002/yea.3979 39262085

[144] Yang X, Gao R, Zhang Q, Yung CCM, Yin H, Li J. 2024. Quantification of polyphosphate in environmental planktonic samples using a novel fluorescence dye JC-D7. Environ Sci Technol 58:14249–14259. doi:10.1021/acs.est.4c04545 39079691 PMC11325646

[145] Schindelin J, Arganda-Carreras I, Frise E, Kaynig V, Longair M, Pietzsch T, Preibisch S, Rueden C, Saalfeld S, Schmid B, Tinevez JY, White DJ, Hartenstein V, Eliceiri K, Tomancak P, Cardona A. 2012. Fiji: an open-source platform for biological-image analysis. Nat Methods 9:676–682. doi:10.1038/nmeth.2019 22743772 PMC3855844

[146] Baggett NS, Bronson AS, Cabeen MT. 2021. SOS-independent pyocin production in P. aeruginosa is induced by XerC recombinase deficiency. MBio 12:e0289321. doi:10.1128/mBio.02893-21 34809462 PMC8609362

[147] Hamm CW, Butler DR, Cabeen MT. 2022. Bacillus subtilis stressosome sensor protein sequences govern the ability to distinguish among environmental stressors and elicit different σ^B^ response profiles. MBio 13:e0200122. doi:10.1128/mbio.02001-22 36409125 PMC9765535

[148] Kulakovskaya EV, Zemskova MY, Kulakovskaya TV. 2018. Inorganic polyphosphate and cancer. Biochemistry (Mosc) 83:961–968. doi:10.1134/S0006297918080072 30208832

