# Supplementary material for: Inorganic polyphosphate and the stringent response coordinately control cell division and cell morphology in Escherichia coli
Source: mBio. 2024 Dec 27;16(2):e03511-24. doi: 10.1128/mbio.03511-24 (PMC11796413; doi:10.1128/mbio.03511-24)
Supplement: Supplemental material — Figures S1 to S16, Table S1, and legends for supplemental videos. [file mbio.03511-24-s0001.pdf]

SUPPLEMENTAL INFORMATION

Inorganic polyphosphate and the stringent response coordinately control cell division and cell morphology in *Escherichia coli*.

Christopher W. Hamm[a] and Michael J. Gray[a,#]

[a]Department of Microbiology, Heersink School of Medicine, University of Alabama at Birmingham, Birmingham, Alabama, USA

Running Head: PolyP and (p)ppGpp regulate cell division and morphology

[#] Address correspondence to Michael J. Gray, mjgray@uab.edu

SUPPLEMENTAL FIGURES

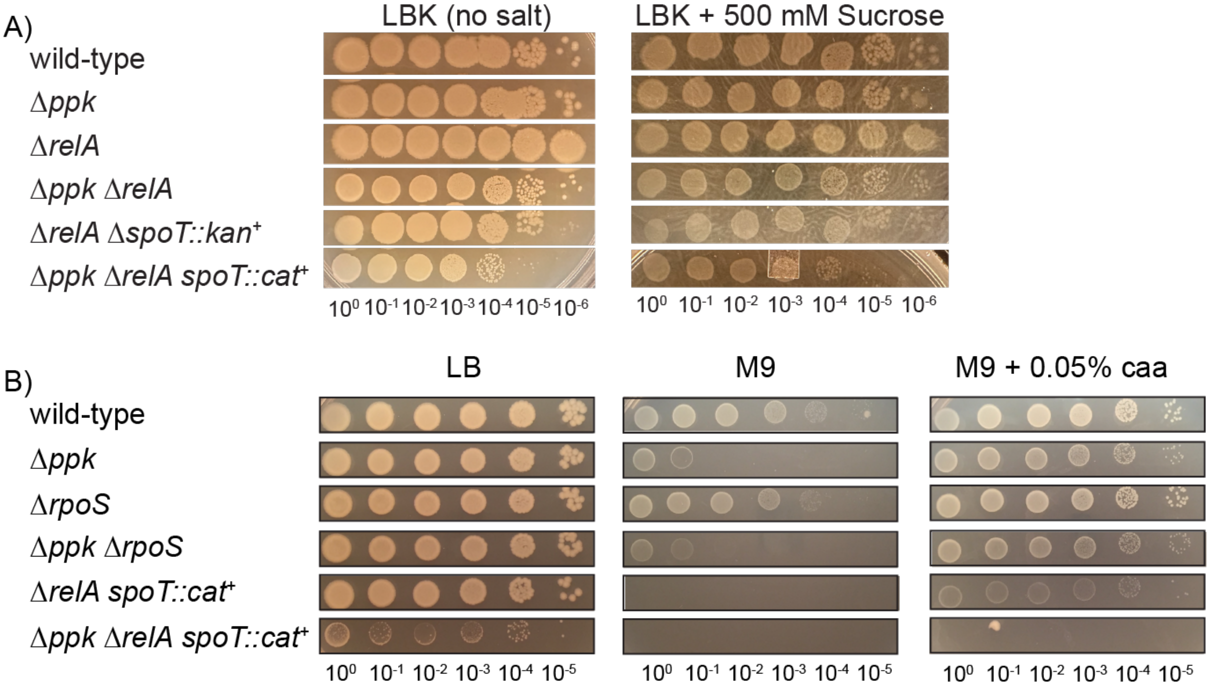

FIG S1 Growth of *ppk, relA* and *spoT* mutants was unaffected by osmotic differences in plates, and deletion of *rpoS* does not phenocopy deletion of *relA* and *spoT* in a *ppk* mutant. A) *E. coli* strains MG1655 (wild-type), MJG0224 (MG1655 Δ*ppk-749*), MJG0226 (MG1655 Δ*relA*), MJG1116 (MG1655 Δ*ppk* Δ*relA*), MJG1287 (MG1655 Δ*relA* Δ*spoT::cat*), MJG1282 (MG1655 Δ*ppk* Δ*relA spoT::cat*) were grown overnight in LB broth, then rinsed and normalized to an A_{600} = 1 in PBS. Aliquots (5 µl) of serially-diluted suspensions were spotted on LBK or LBK + 500 mM sucrose plates and then incubated overnight at 37°C (representative image from at least 3 independent experiments). **B)** *E. coli* strains MG1655 (wild-type), MJG0224 (MG1655 Δ*ppk-749*), MJG0344 (MG1655 Δ*rpoS746*), MJG1119 (MG1655 Δ*rpoS746* Δ*relA782*), MJG1136 (MG1655 Δ*relA782 spoT207*::*cat*$^{+}$), and MJG1137 (MG1655 Δ*ppk-749* Δ*relA782 spoT207*::*cat*$^{+}$) were grown overnight in LB broth, then rinsed and normalized to an A_{600} = 1 in PBS. Aliquots (5 µl) of serially-diluted suspensions were spotted on LB, M9

glucose, or M9 glucose containing 0.05% (w/v) casamino acids (c.a.a.) plates and incubated overnight at 37°C (representative image from at least 3 independent experiments).

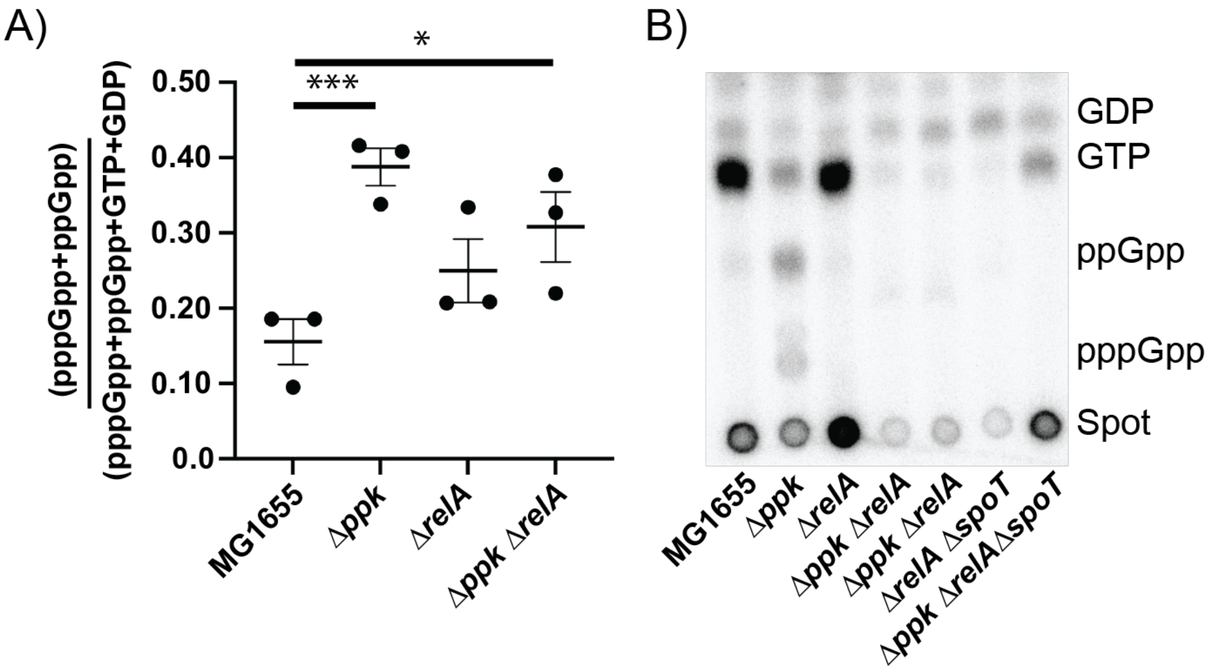

FIG S2 Mutants lacking polyP accumulate excess (p)ppGpp in minimal medium.

A) Fraction of (p)ppGpp in *E. coli* cells grown in MOPS minimal media quantified by growing cells in the presence of radioactive ^{32}P (Phosphorus-32 Radionuclide, 1mCi (37 MBq) ®Revvity). Cells were grown for 2-3 doublings in the presence of ^{32}P, and then harvested and run on TLC plate and imaged using a Typhoon Biomolecular Imager, with intensity quantified using ImageQuant. Normalization was calculated by (pppGpp + ppGpp)/(pppGpp + ppGpp + GTP + GDP) (1-4) for the fraction of (p)ppGpp present in the cell. * = P-value < 0.05, *** = P-value < 0.0005, statistical analysis performed in Prism via one-way ANOVA, quantified in triplicate. **B)** Representative image of TLC plate from MOPS minimal media growth experiment.

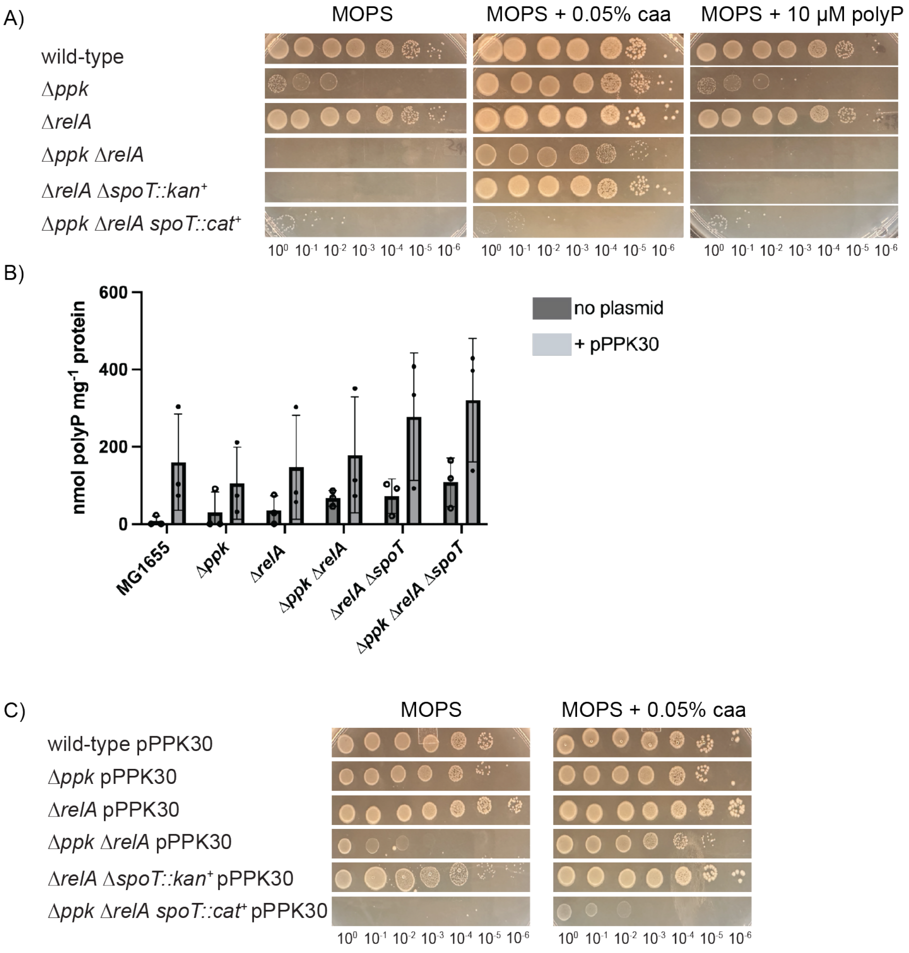

FIG S3 The impact of either exogenous or increased intracellular polyP on growth of *E. coli* strains on minimal media. A) *E. coli* strains MG1655 (wild-type), MJG0224 (MG1655 Δ*ppk-749*), MJG0226 (MG1655 Δ*relA*), MJG1116 (MG1655 Δ*ppk* Δ*relA*), MJG1287 (MG1655 Δ*relA* Δ*spoT::cat*), MJG1282 (MG1655 Δ*ppk* Δ*relA spoT::cat*) were grown overnight in LB broth, then rinsed and normalized to an A_{600} = 1 in PBS. Aliquots (5 µl) of serially-diluted suspensions were spotted on MOPS glucose plates with or without 0.05% casamino acids or 10 µM polyP (Acros organics) and then incubated overnight at 37°C (representative image from at least 3 independent experiments). **B**) Quantification of polyP in overnight LB broth cultures of *E. coli* strains MG1655 (MJG0001), Δ*ppk* (MJG0224), Δ*relA* (MJG0226), Δ*ppk* Δ*relA* (MJG1116), Δ*relA* Δ*spoT*

(MJG1287), and Δ*ppk* Δ*relA* Δ*spoT* (MJG1282), as well as MG1655 / pPPK30 (*ppk*$^{\mathrm{G733A}}$ *bla*$^{+}$)(MJG2577), Δ*ppk* / pPPK30 (*ppk*$^{\mathrm{G733A}}$ *bla*$^{+}$)(MJG2578), Δ*relA* / pPPK30 (*ppk*$^{\mathrm{G733A}}$ *bla*$^{+}$)(MJG2579), Δ*ppk* Δ*relA* / pPPK30 (*ppk*$^{\mathrm{G733A}}$ *bla*$^{+}$)(MJG2580), Δ*relA* Δ*spoT* / pPPK30 (*ppk*$^{\mathrm{G733A}}$ *bla*$^{+}$)(MJG2581), and Δ*ppk* Δ*relA* Δ*spoT* / pPPK30 (*ppk*$^{\mathrm{G733A}}$ *bla*$^{+}$)(MJG2582). **C**) *E. coli* strains MJG2577 (MG1655 pPPK30 (*ppk*^{G733A} *bla*$^{+}$)), MJG2578 (Δ*ppk-749* pPPK30 (*ppk*^{G733A} *bla*$^{+}$)), MJG2579 (ΔrelA pPPK30 (*ppk*^{G733A} *bla*$^{+}$)), MJG2580 (Δ*ppk-749* Δ*relA782*::*kan*$^{+}$ pPPK30 (*ppk*^{G733A} *bla*$^{+}$)), MJG2581 (Δ*relA782* Δ*spoT1000*::*kan*$^{+}$ pPPK30 (*ppk*^{G733A} *bla*$^{+}$)), or MJG2582 (Δ*ppk-749* Δ*relA782* Δ*spoT1000*::*kan*$^{+}$ pPPK30 (*ppk*^{G733A} *bla*$^{+}$)) were grown overnight in LB broth, then rinsed and normalized to an A_{600} = 1 in PBS. Aliquots (5 µl) of serially-diluted suspensions were spotted on MOPS or MOPS containing 0.05% (w/v) casamino acids (c.a.a.) plates and incubated overnight at 37°C (representative image from at least 3 independent experiments).

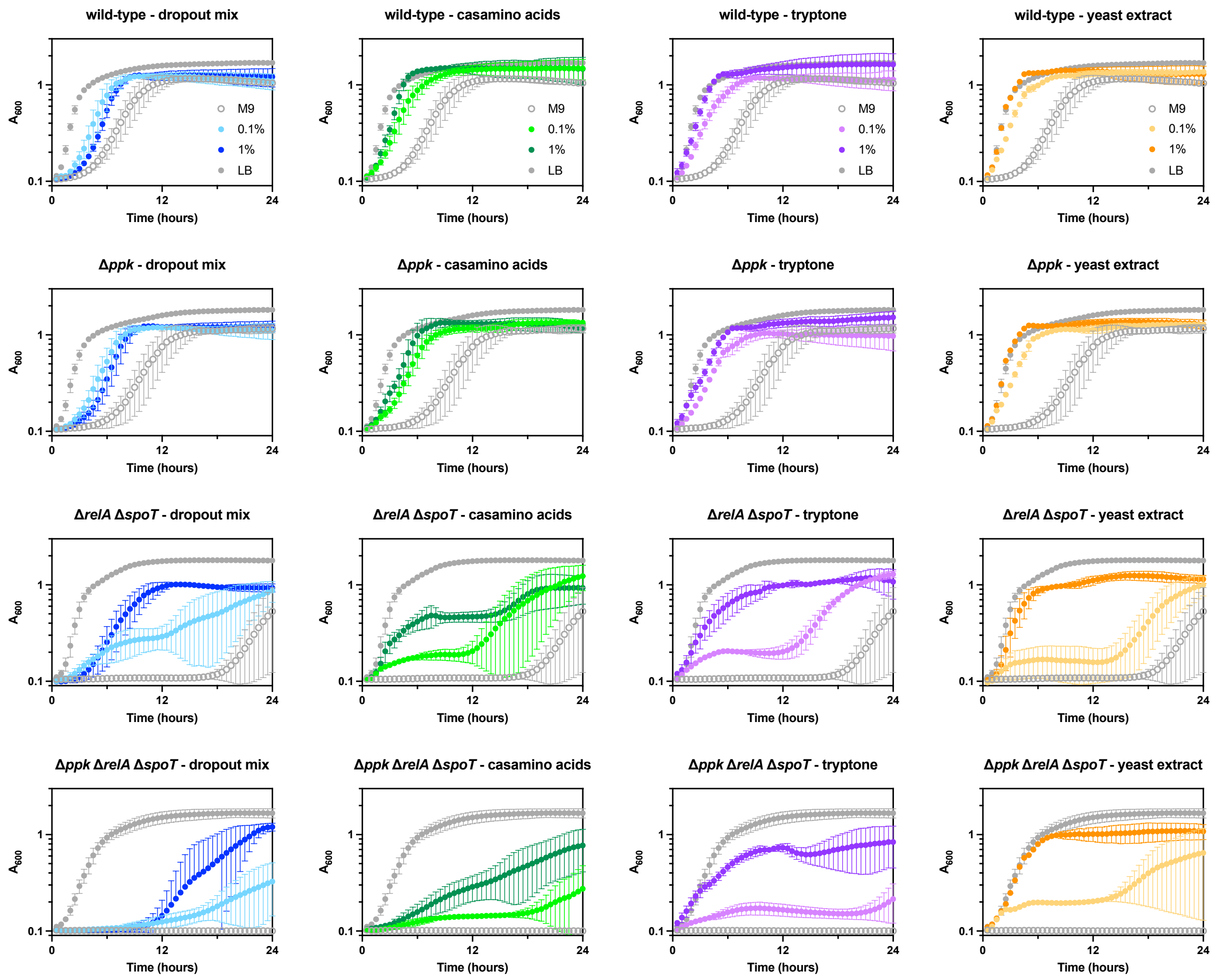

FIG S4 Effect of media supplements on growth of *ppk relA spoT* mutants in liquid media. *Escherichia coli* strains MG1655, MJG0224 (MG1655 Δ*ppk-749*), MJG1287 (MG1655 Δ*relA782* Δ*spoT1000*::*kan*$^+$), and MJG1282 (MG1655 Δ*ppk-749* Δ*relA782* Δ*spoT1000*::*kan*$^+$) were grown overnight at 37°C with shaking in Lysogeny Broth (LB), then normalized to a A_{600} = 1 and rinsed three times with sterile PBS. The resulting cell suspensions were diluted 1:40 into LB or M9 minimal medium containing 0.4% glucose and 100 µM $FeCl_3$ with or without supplementation with 0.1% or 1% (w/v) of yeast synthetic dropout mix supplement, casamino acids, tryptone, or yeast extract. Growth curves were performed in clear 96-well plates in a Tecan Spark plate reader, incubating

at 37°C with shaking and measuring A_{600} at 30-minute intervals for 24 hours (n=3-6 experimental replicates, with 3-12 technical replicates per experiment).

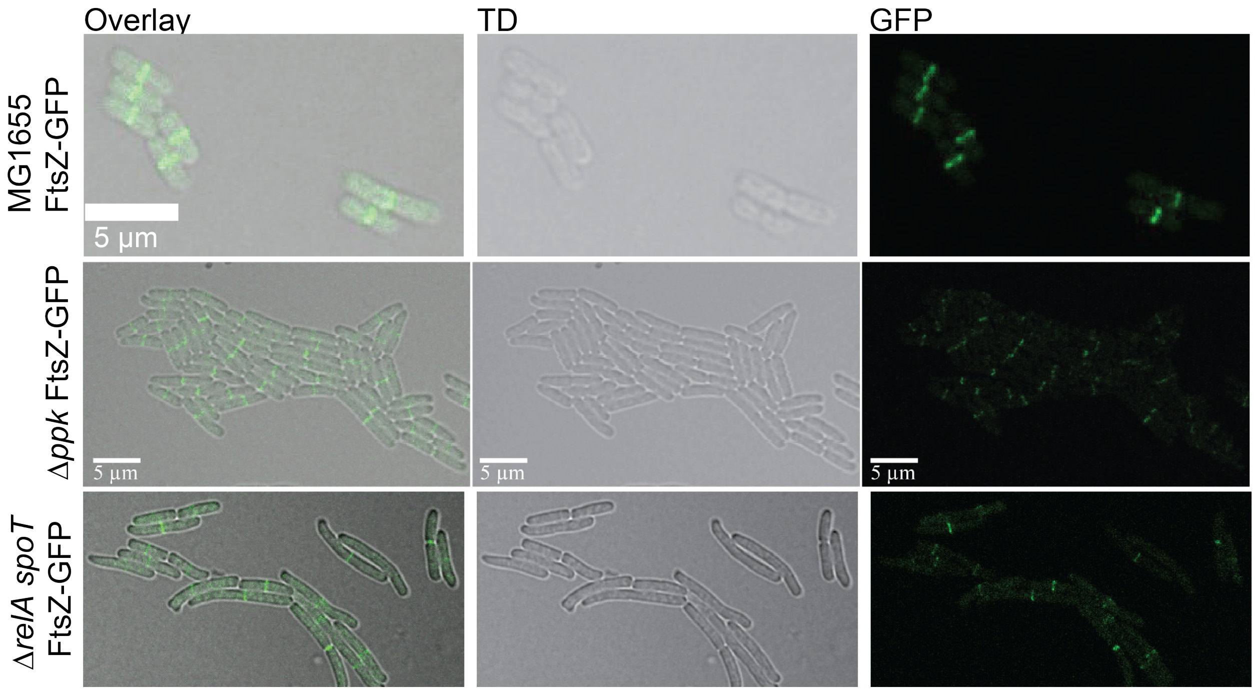

FIG S5 Representative images of FtsZ reporter strains during microscopy. Here is a representative image of MG1655 FtsZ-GFP (MJG 2401), Δ*ppk* FtsZ-GFP (MJG2402) and Δ*relA spoT* FtsZ-GFP (MJG2404) grown on LB agarose pads at 37°C and imaged in TD and GFP on a confocal microscope. FtsZ rings appear at the midpoint of most cells, with a single FtsZ ring per cell, which also occurs for all single and double *ppk*, *relA* and *spoT* mutants (data not shown).

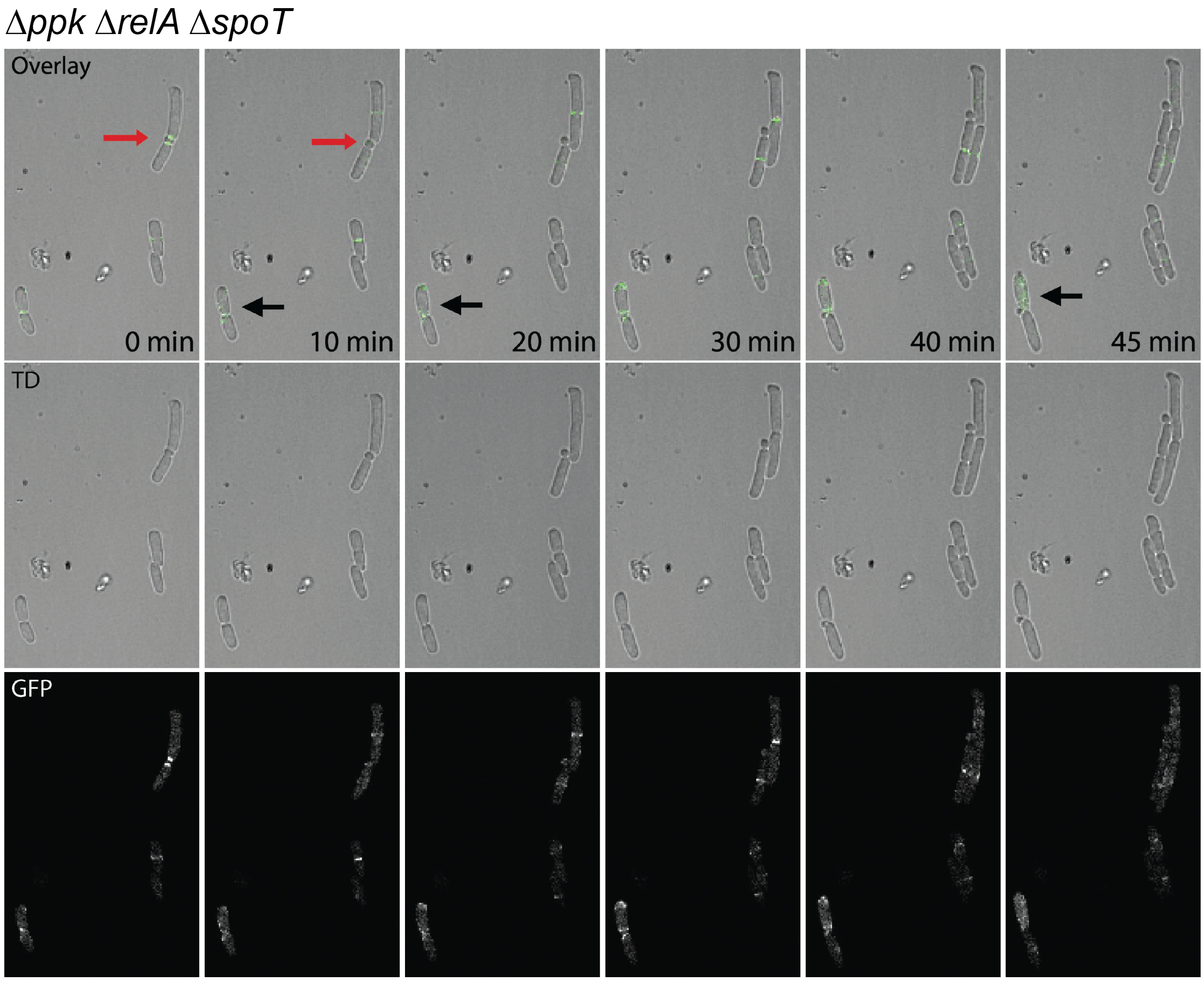

Fig S6 Strains lacking polyP and (p)ppGpp have disrupted cell division. (These data are the separated channels for **Fig 5A**, provided for clarity.) Confocal fluorescence time-lapse microscopy of the mutant *ppk relA spoT* FtsZ-GFP (MJG2405) on an LB agarose pad at 37°C, illustrating mini cell production by this strain. In one case, the triple mutant forms two Z-rings in the middle of the cell, releasing a mini cell (red arrows). There are also two Z-rings which form at either pole of a single cell, both functional and releasing a mini cell (black arrows).

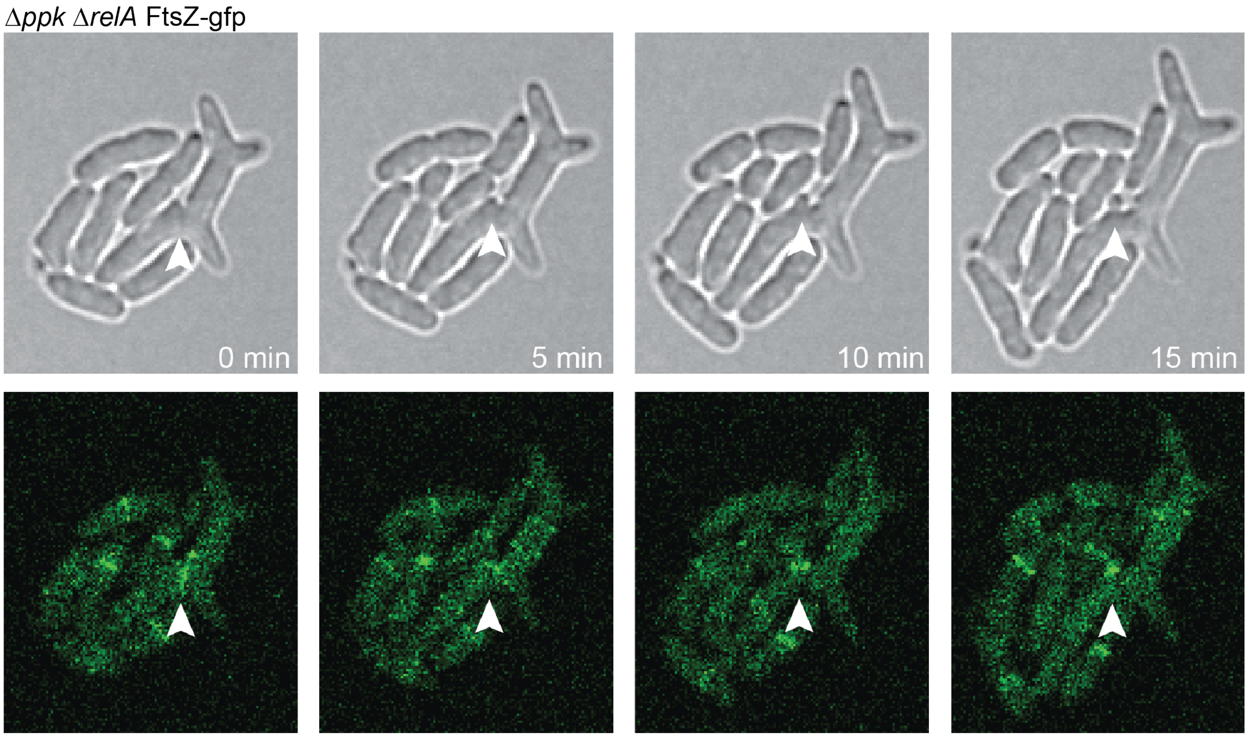

FIG S7 FtsZ ring forming incorrectly, releasing a non-functional mini-cell from a branched cell in a *ppk relA* mutant. *E. coli* MG1655 mutant strain *ppk relA* FtsZ-gfp (MJG2403) growing on a LB agarose pad at 37°C, imaged in GFP during time-lapse fluorescent microscopy. The image here is of a branching cell where we see the FtsZ ring forming in the branch junction (white arrow) before moving to the side wall of the cell and releasing a mini-cell.

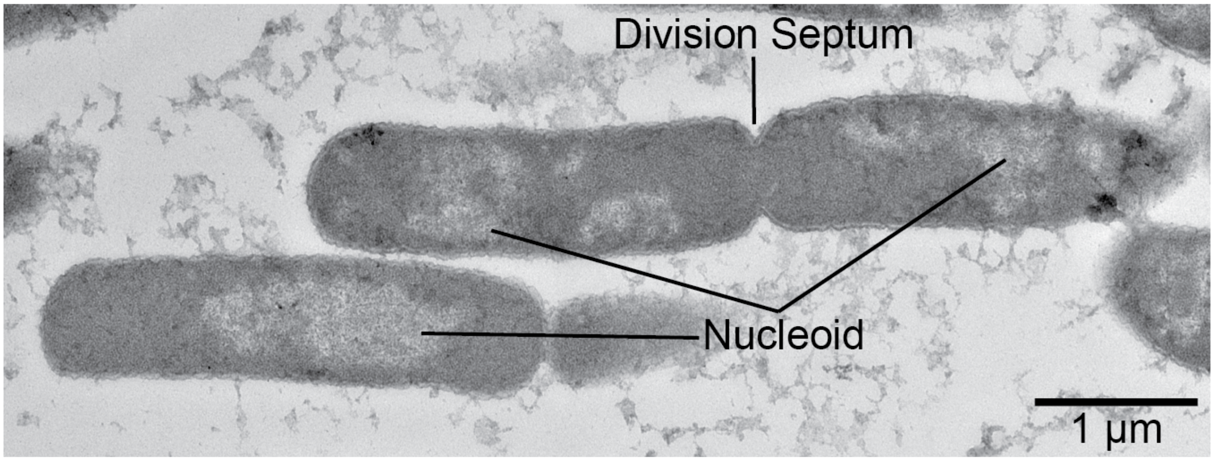

FIG S8 TEM of normal cellular division in the wild type MG1655. *E. coli* MG1655 (MJG0001) dividing at the mid-point of bacterial cell. Cells were grown in LB to exponential phase, OD_{600} = 0.2, before being centrifuged and resuspended in fixative. Here you can see normal cell division occurring, with the nucleoid separated by the poles in the dividing cell.

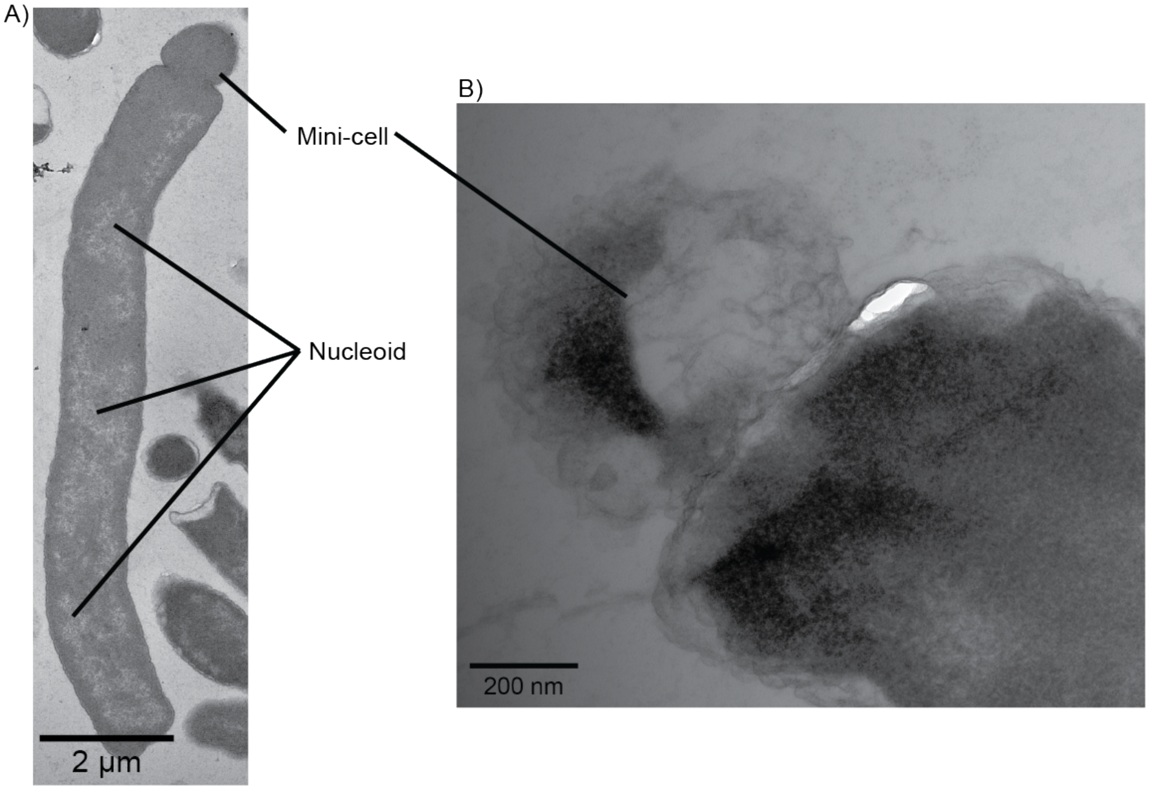

FIG S9 TEM images of *ppk relA spoT* cells filamenting and releasing mini-cells from the pole of the cell. A) Here is a TEM image of *ppk relA spoT* (MJG1282) filamentous cell which is releasing a mini-cell from the pole of the cell. The nucleoid appears to be spread out throughout the filamentous cell, with the exception of the mini-cell. There appears to be no DNA within the mini-cell. **B)** This is a close up image of a different mini-cell forming from the pole of a *ppk relA spoT* (MJG1282) cell.

MG1655

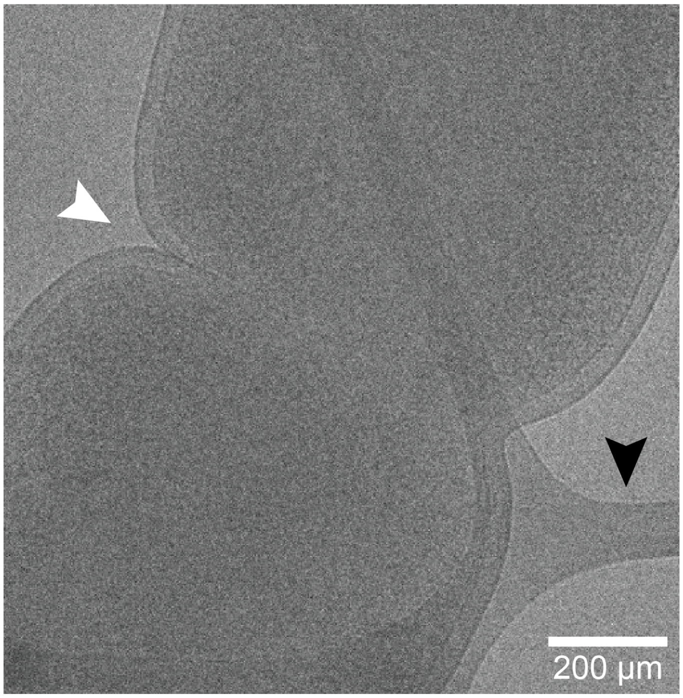

FIG S10 Cryo-electron microscopy of normal cellular division in the wild type MG1655. *E. coli* MG1655 (MJG0001) dividing at the mid-point of bacterial cell. Cells were grown in LB to exponential phase OD_{600} = 0.1 before being centrifuged and resuspended in sterile PBS media before prep for CEM. Black arrows denote the carbon lattice upon which the cells are suspended for CEM. White arrow denotes site of cellular division.

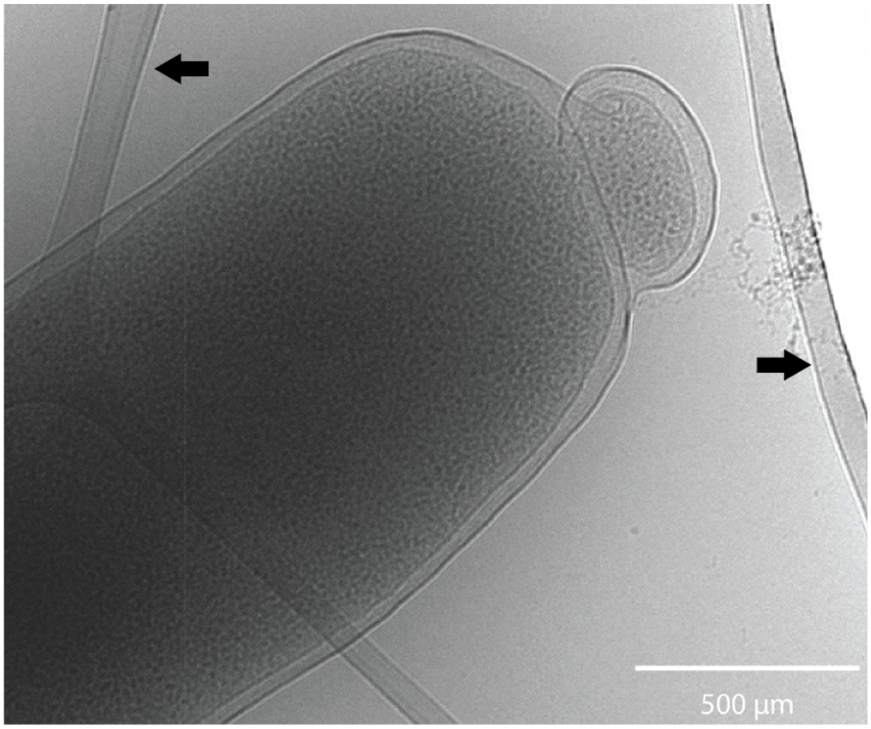

FIG S11 Cryo-electron microscopy of a mini-cell formation from aberrant FtsZ ring placement. Δ*ppk* Δ*relA* Δ*spoT* mutant (MJG2405) forming a mini-cell at a pole. Cells were grown in LB to exponential phase OD_{600} = 0.1 before being centrifuged and resuspended in sterile PBS media before prep for CEM. Black arrows denote the carbon lattice upon which the cells are suspended for CEM.

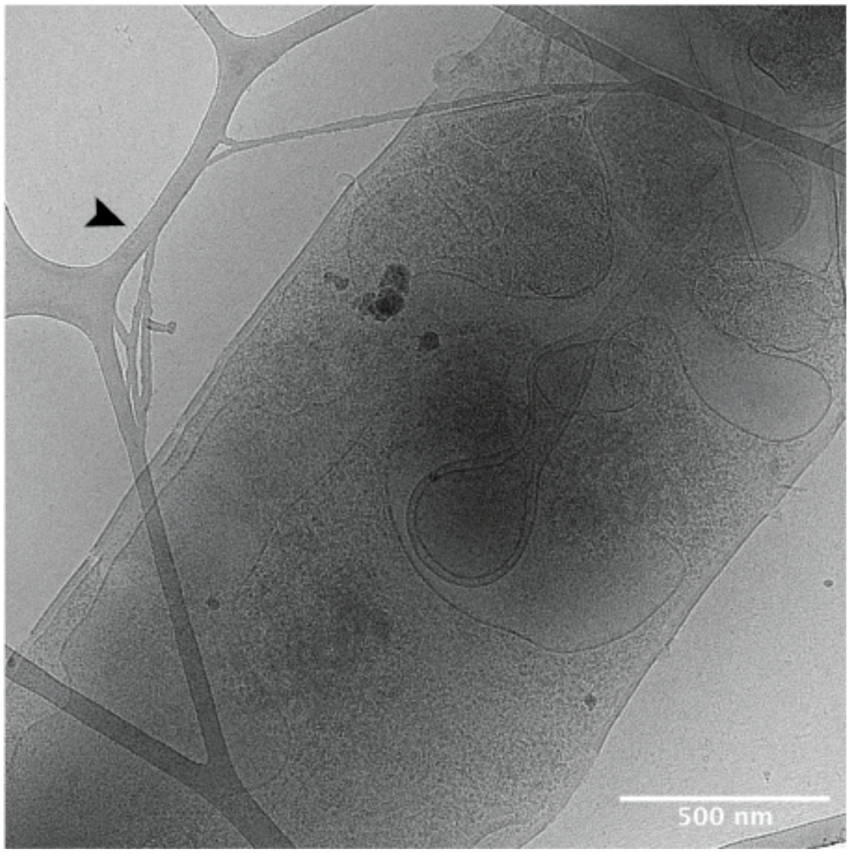

FIG S12 Cryo-electron microscopy showing cytoplasmic condensation in the triple mutant. In this cell of the *ppk relA spoT* mutant (MJG2405), we can see cytoplasmic condensation as well as what appear to be holes in the outer membrane of the cell. Cells were grown in LB to exponential phase OD_{600} = 0.1 before being centrifuged and resuspended in sterile PBS media before prep for CEM. Black arrows denote the carbon lattice upon which the cells are suspended for CEM.

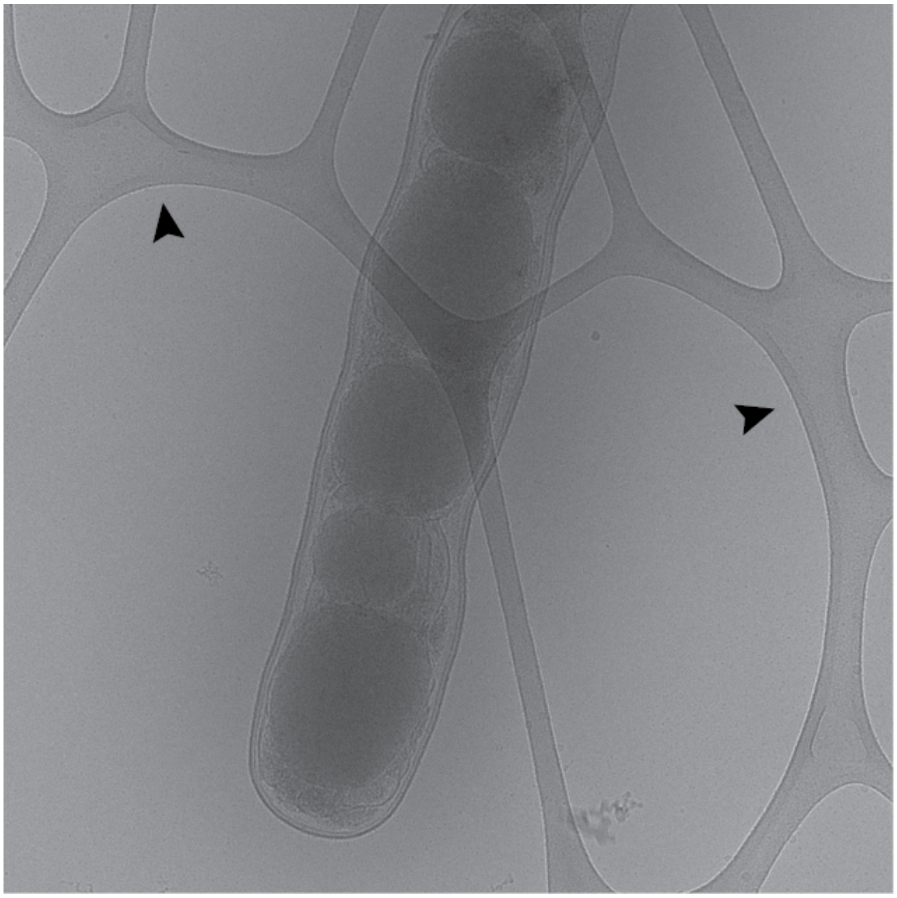

FIG S13 Cryo-electron microscopy showing cytoplasmic condensation after desiccation showing an "edamame" phenotype. In this cell of the Δ*ppk* Δ*relA* Δ*spoT* mutant (MJG2405), we can see cytoplasmic condensation where the cytoplasmic contents have aggregated in small collections within the cell, pulling away from the outer membrane. Cells were grown in LB to exponential phase OD_{600} = 0.1 before being centrifuged and resuspended in sterile PBS media before prep for CEM. Black arrows denote the carbon lattice upon which the cells are suspended for CEM.

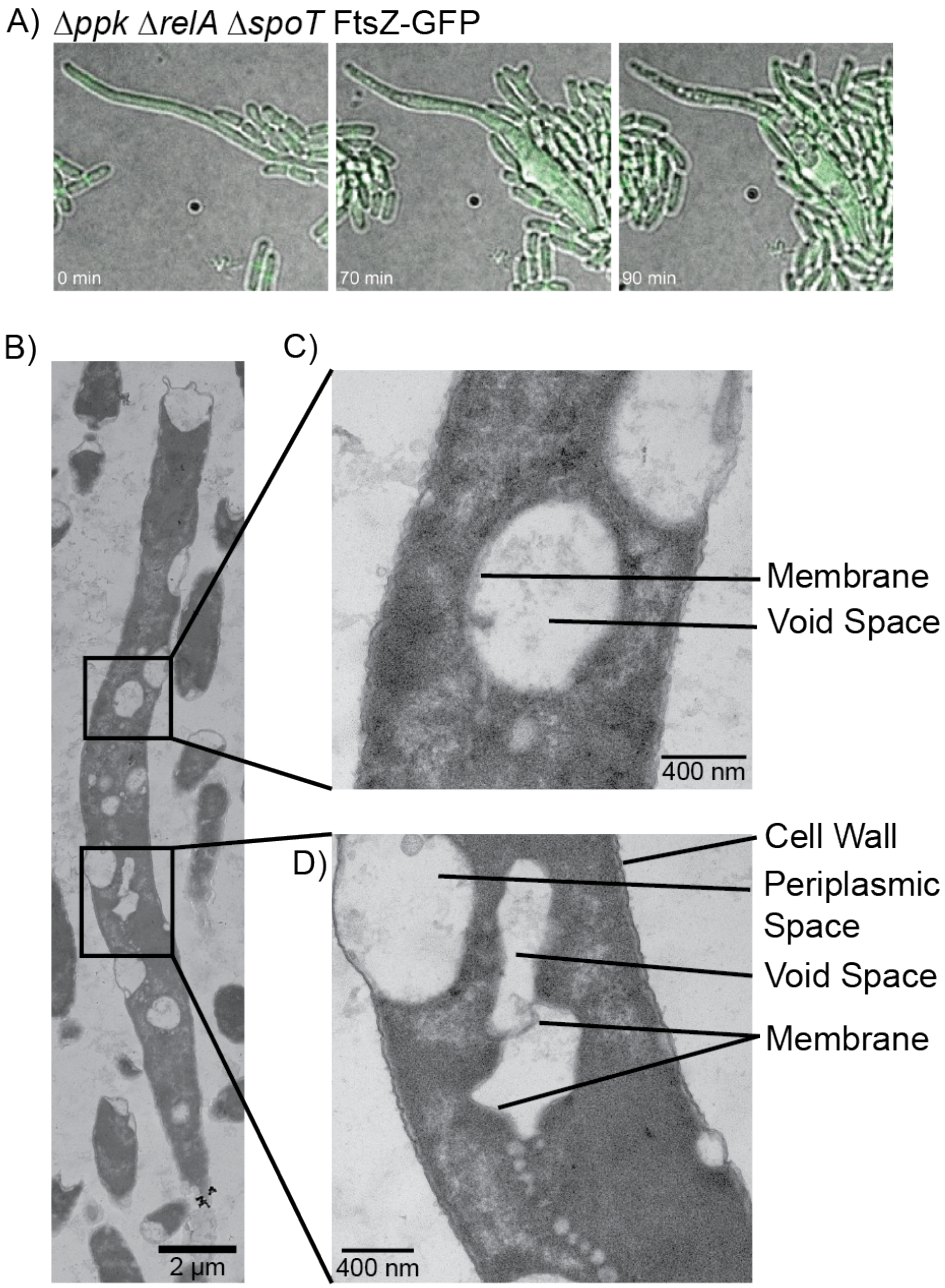

FIG S14 Confocal fluorescence microscopy and transmission electron microscopy showing cells with multiple "holes" showing a Swiss cheese like phenotype. A) Here is a time-lapse microscopy *ppk relA spoT* FtsZ-GFP (MJG2405) grown on LB agarose pads at 37°C and imaged in TD and GFP on a confocal microscope. This cell develops into a filamentous cell, which then bulges and develops

holes, which we theorize represents cytoplasmic condensation from leaking cytoplasmic contents. **B)** TEM of *ppk relA spoT* (MJG1282) showing multiple “holes” or void spaces throughout the cell. **C)** This is a closer look at the cell highlighted by the associated box in panel B), in which we can see what appears to be an intact membrane surrounding the void space within the cell. **D)** In this zoomed in section of the cell we can see what appear to membranes surrounding and folded in between two separate void spaces. This suggests these are separate compartments.

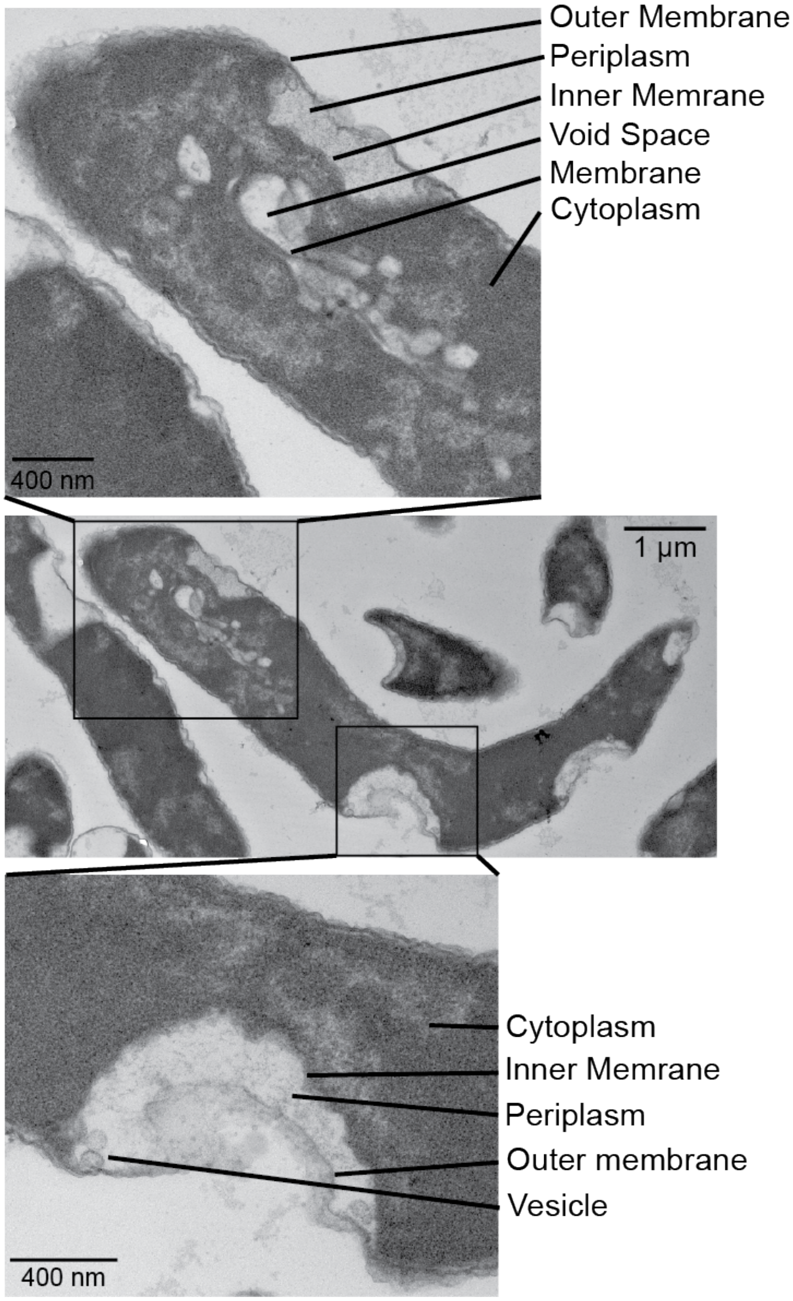

FIG S15 TEM of a *ppk relA spoT* mutant. This cell appears to have many small compartments or void spaces separated by membranes (top image), as well as many large periplasmic spaces (top and bottom images). The inner membrane appears to be separating away from the cell wall creating a large periplasmic space.

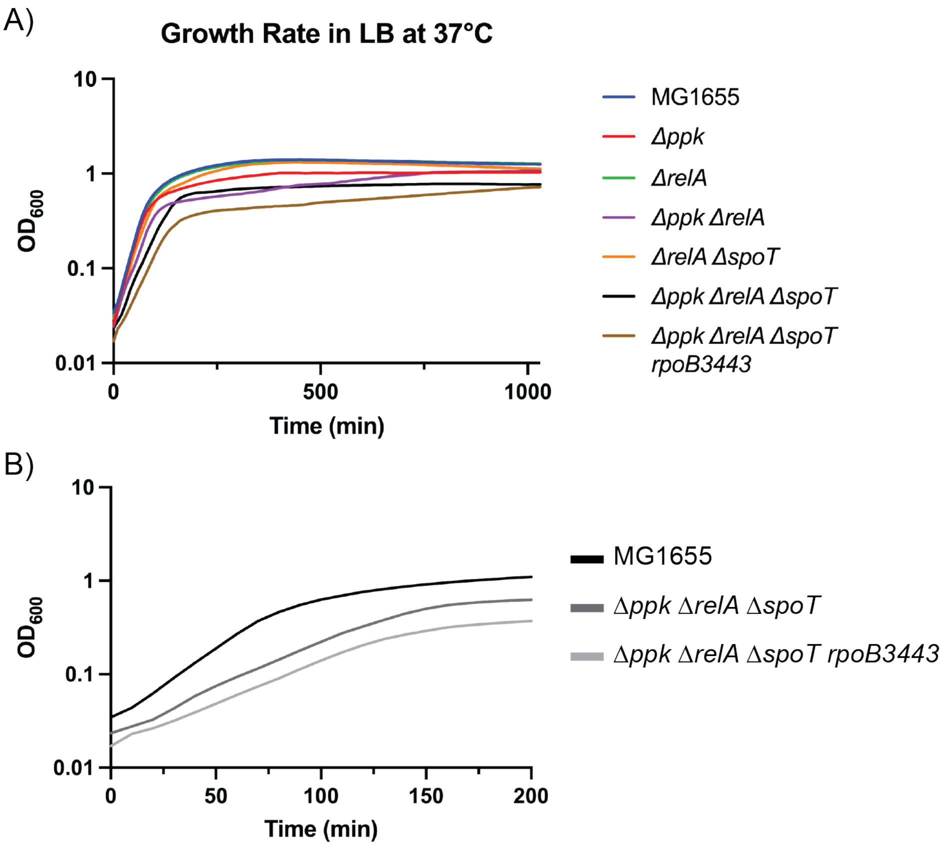

C)

Strain	Doubling Time (min)	CI (95%)
MG1655	20.25	15.67 - 27.30
Δppk ΔrelA ΔspoT	33.57	29.00 - 39.49
Δppk ΔrelA ΔspoT rpoB3443	35.44	23.85 - 38.35

FIG S16 Growth curve comparison of mutants. A) Growth curve of *E. coli* strains wild type (MJG0001), *ppk* (MJG0224), *relA* (MJG0226), *ppk relA* (MJG1116), *relA spoT* (MJG1287), *ppk relA spoT* (MJG1282), and *ppk relA spoT rpoB3443* (MJG1579) were grown in LB shaking at 37°C for 24 hours. Panel (**B**) shows the first 200 minutes of the growth curve for MG1655, MJG1282, and MJG1579, calculated doubling times from the peak rate of growth for each strain below (**C**). All statistics and calculations were performed in GraphPad Prism.

Supplemental Table 1: Statistical analysis of cell lengths and percent of cells branching, with disrupted Z-rings and plasmolysis, as well as FtsZ positioning.

2-Way ANOVA comparison of cell lengths in MOPS minimal Media

Tukey's multiple comparisons test	Mean Diff.	95.00% CI of diff.	Below threshold?	Summary	Adjusted P Value
MG1655 vs. Δppk	-0.19	-0.4118 to 0.02797	No	ns	0.12
MG1655 vs. ΔrelA	-0.25	-0.4606 to -0.03655	Yes	*	0.01
MG1655 vs. Δppk ΔrelA	-0.23	-0.4551 to -0.003176	Yes	*	0.05
MG1655 vs. ΔrelA ΔspoT	-0.91	-1.133 to -0.6940	Yes	****	<0.0001
Δppk vs. ΔrelA	-0.06	-0.2780 to 0.1646	No	ns	0.96
Δppk vs. Δppk ΔrelA	-0.04	-0.2719 to 0.1975	No	ns	0.99
Δppk vs. ΔrelA ΔspoT	-0.72	-0.9504 to -0.4931	Yes	****	<0.0001
ΔrelA vs. Δppk ΔrelA	0.02	-0.2079 to 0.2468	No	ns	1.00
ΔrelA vs. ΔrelA ΔspoT	-0.67	-0.8862 to -0.4440	Yes	****	<0.0001
Δppk ΔrelA vs. ΔrelA ΔspoT	-0.68	-0.9190 to -0.4500	Yes	****	<0.0001

	**MG1655**	**Δppk**	**ΔrelA**	**Δppk ΔrelA**	**ΔrelA ΔspoT**
**n**	365.00	309.00	355.00	280.00	310.00
**Mean**	2.50	2.69	2.75	2.73	3.42
Std. Deviation	0.66	0.94	1.32	0.81	1.29
Std. Error of Mean	0.03	0.05	0.07	0.05	0.07

2- Way ANOVA comparison of cell length in LB

Tukey's multiple comparisons test	Mean Diff.	95.00% CI of diff.	Below threshold?	Summary	Adjusted P Value
MG1655 vs. Δ*ppk*	-1.58	-3.483 to 0.3232	No	ns	0.17
MG1655 vs. Δ*ppk* Δ*relA*	-4.28	-6.115 to -2.444	Yes	****	<0.0001
MG1655 vs. Δ*relA* Δ*spoT*	-5.08	-6.943 to -3.221	Yes	****	<0.0001
MG1655 vs. Δ*ppk* Δ*relA* Δ*spoT*	-2.03	-3.866 to -0.1959	Yes	*	0.02
MG1655 vs. Δ*relA*	-7.29	-9.142 to -5.445	Yes	****	<0.0001
Δ*ppk* vs. Δ*ppk* Δ*relA*	-2.70	-4.544 to -0.8549	Yes	***	0.00
Δ*ppk* vs. Δ*relA* Δ*spoT*	-3.50	-5.373 to -1.632	Yes	****	<0.0001
Δ*ppk* vs. Δ*ppk* Δ*relA* Δ*spoT*	-0.45	-2.296 to 1.394	No	ns	0.98
Δ*ppk* vs. Δ*relA*	-5.71	-7.572 to -3.855	Yes	****	<0.0001
Δ*ppk* Δ*relA* vs. Δ*relA* Δ*spoT*	-0.80	-2.581 to 0.9751	No	ns	0.79
Δ*ppk* Δ*relA* vs. Δ*ppk* Δ*relA* Δ*spoT*	2.25	0.5396 to 3.957	Yes	**	0.00
Δ*ppk* Δ*relA* vs. Δ*relA*	-3.01	-4.766 to -1.261	Yes	****	<0.0001
Δ*relA* Δ*spoT* vs. Δ*ppk* Δ*relA* Δ*spoT*	3.05	1.273 to 4.829	Yes	****	<0.0001
Δ*relA* Δ*spoT* vs. Δ*relA*	-2.21	-4.003 to -0.4189	Yes	**	0.01
Δ*ppk* Δ*relA* Δ*spoT* vs. Δ*relA*	-5.26	-7.015 to -3.510	Yes	****	<0.0001

	MG1655	*Δppk*	*ΔrelA*	*Δppk ΔrelA*	*ΔrelA ΔspoT*
**n**	315.00	310.00	362.00	510.00	347.00
**Mean**	3.12	4.69	10.96	8.72	8.55
Std. Deviation	0.77	2.09	13.07	7.88	9.15
Std. Error of Mean	0.04	0.12	0.69	0.35	0.49

	Δppk ΔrelA ΔspoT
**n**	387.00
**Mean**	6.40
Std. Deviation	10.24
Std. Error of Mean	0.52

Number of cells with plasmolysis

Strain	Total cells	Cells with Plasmolysis	Percent of cells with plasmolysis
Δppk ΔrelA ΔspoT (MJG 1282)	1794.00	20.00	1.1%

Number of cells with branching

Strain	Total cells	Cells with Branching	Percent of cells with branching
Δppk ΔrelA (MJG1116)	2276.00	43.00	1.9%
Δppk ΔrelA ΔspoT (MJG1282)	3081.00	68.00	2.2%

z between *Δppk ΔrelA* and *Δppk ΔrelA ΔspoT* is -0.8072. The value of *p* is .41794

Number of cells with Disrupted Z-rings

Strain	Total cells	Cells with disrupted Z-ring	Percent of cells with Disrupted Z-rings
Δppk ΔrelA (MJG1116)	1241.00	58.00	4.7%
Δppl ΔrelA ΔspoT (MJG1282)	3081.00	25.00	0.8%

z between Δppk ΔrelA and Δppk ΔrelA ΔspoT is 8.3703. The value of *p* is < .00001. The result is significant at *p* < .05.

% of shrinking of *Δppk ΔrelA ΔspoT* when grown on MOPS minimal media over 3 hours

Cell	Original size	Size after MOPS	% shrinkage
1.00	4.30	3.92	8.9%
2.00	3.20	2.65	17.0%
3.00	5.13	4.81	6.3%
4.00	8.94	6.89	22.9%
5.00	4.92	4.25	13.8%
6.00	6.51	6.13	5.8%
7.00	4.84	4.83	0.2%
8.00	4.25	4.03	5.3%
9.00	2.93	2.75	5.9%
10.00	5.15	4.75	7.9%

11.00	8.82	6.92	21.6%
12.00	4.96	4.54	8.5%
13.00	6.29	5.34	15.2%
14.00	8.00	6.65	16.8%
15.00	4.55	3.70	18.7%
16.00	5.22	4.52	13.4%
17.00	4.35	2.90	33.3%
18.00	3.52	2.85	19.0%
19.00	4.12	3.15	23.6%
20.00	4.46	3.84	13.8%
21.00	4.90	4.25	13.3%
22.00	5.53	5.22	5.6%
23.00	3.08	2.61	15.1%
24.00	3.10	2.56	17.4%
25.00	9.00	8.00	11.1%
26.00	3.98	3.04	23.6%
27.00	5.14	4.96	3.5%
28.00	7.77	6.57	15.5%
29.00	4.32	3.69	14.5%
30.00	5.81	4.57	21.3%
31.00	3.20	2.28	28.7%
32.00	4.34	3.00	30.9%
33.00	5.78	4.86	15.9%
34.00	12.75	10.61	16.8%
35.00	6.92	6.08	12.1%
36.00	4.62	3.56	23.0%
37.00	9.21	7.35	20.2%
			**15.3%**

∆ppk ∆relA ∆spoT shrinks an average of **15.31%** once plated on MOPS minimal media.

SUPPLEMENTAL VIDEOS

Supplemental Video 1. Time-lapse fluorescent microscopy of wild-type MG1655 (MJG0001) on MOPS minimal media agarose pad at 37°C.

Supplemental Video 2. Time-lapse fluorescent microscopy of *ppk relA* mutant (MJG2403) with FtsZ-GFP reporter on MOPS minimal media agarose pad at 37°C.

Supplemental Video 3. Time-lapse fluorescent microscopy of *ppk relA spoT* mutant (MJG2405) with FtsZ-GFP reporter on MOPS minimal media agarose pad which no longer grows and shrinks over time 37°C.

Supplemental Video 4. Time-lapse fluorescent microscopy of wild-type MG1655 (MJG2401) with FtsZ-GFP reporter on LB media agarose pad at 37°C.

Supplemental Video 5. Time-lapse fluorescent microscopy of *ppk relA* FtsZ-GFP (MJG2403) mutant on LB agarose pad at 37°C. This video has six Z-rings within a single growing cell. **Fig 5B** is a still image from this video.

Supplemental Video 6. Time-lapse fluorescent microscopy of *ppk relA* FtsZ-GFP (MJG2403) mutant on LB agarose pad at 37°C showing branching cells developing, as shown in still images in **Fig 7**.

Supplemental Video 7. Time-lapse fluorescent microscopy of *ppk relA spoT* FtsZ-GFP (MJG2405) mutant on LB agarose pad at 37°C showing branching cells developing.

Supplemental Video 8. Time-lapse fluorescent microscopy of *ppk relA spoT* FtsZ-GFP (MJG2405) mutant on LB agarose pad at 37°C showing branching cells and a spheroplast developing in the lower left corner of the video.

Supplemental Video 9. Time-lapse microscopy of Δ*ppk* Δ*relA* Δ*spoT rpoB3443* (MJG1581) on LB agarose pad at 37°C, imaged every 10 minutes.

SUPPLEMENTAL REFERENCES

1. Schneider DA, Gourse RL. 2004. Relationship between growth rate and ATP concentration in Escherichia coli: a bioassay for available cellular ATP. J Biol Chem 279:8262-8.
2. Liu K, Bittner AN, Wang JD. 2015. Diversity in (p)ppGpp metabolism and effectors. Curr Opin Microbiol 24:72-9.
3. Spira B, Ospino K. 2020. Diversity in E. coli (p)ppGpp Levels and Its Consequences. Front Microbiol 11:1759.
4. Roghanian M, Van Nerom K, Takada H, Caballero-Montes J, Tamman H, Kudrin P, Talavera A, Dzhygyr I, Ekstrom S, Atkinson GC, Garcia-Pino A, Hauryliuk V. 2021. (p)ppGpp controls stringent factors by exploiting antagonistic allosteric coupling between catalytic domains. Mol Cell 81:3310-3322 e6.
